# Phylogenetic network of infectious bronchitis virus: exploring the impact of migratory birds on viral clustering, evolution, and recombination

**DOI:** 10.1080/01652176.2025.2465570

**Published:** 2025-02-14

**Authors:** Yu-Chen Tai, Geng-Ming Hu, Chi-Ming Chen

**Affiliations:** Department of Physics, National Taiwan Normal University, Taipei, Taiwan

**Keywords:** Infectious bronchitis virus (IBV), phylogenetic network, MSClustering, migratory birds, recombination events

## Abstract

Infectious bronchitis virus (IBV) presents a major threat to global poultry production, necessitating a thorough understanding of its evolutionary relationships for effective control measures. This study presents a novel distance-based Minimum Span Clustering (MSClustering) method to cluster 311 IBV strains, with a comparison of its results to the established IBV classification. Phylogenetic network and recombination analyses were employed to investigate IBV evolutionary relationships and transmission pathways. The phylogenetic network revealed distinct clusters reflecting relationships between IBV strains. Importantly, these network patterns, combined with recombination event analysis, suggest an unrecognized role for migratory birds in IBV dissemination, highlighting potential transmission pathways beyond established poultry trade routes. These findings contribute to advancing our understanding of IBV evolution and support the development of targeted strategies for controlling viral outbreaks in poultry populations. While statistical limitations may affect threshold estimation for smaller networks, our MSClustering method significantly accelerates processing speeds—approximately 100,000 times faster than PhyML when analyzing the dataset—enabling comprehensive-scale phylogenetic analysis of viruses.

## 1. Introduction

Infectious bronchitis virus (IBV) poses a significant and persistent challenge in poultry farming, ranking as the second most costly poultry disease globally (World Bank and TAFS Forum 2011). IBV’s ability to rapidly spread among flocks, leading to various clinical symptoms, has posed significant challenges for poultry farmers and veterinarians alike. Despite decades of research and control efforts, IBV continues to evolve, leading to the emergence of new strains and genotypes that perplex our understanding of this pathogen. Since 2016, new genotypes and lineages such as GI-28, GI-29, and GVIII in China, GI-30 in Trinidad and Tobago, GI-31 and GIX in Mexico, and GII-2 and GVII-2 in Europe have been identified across various regions worldwide (Abozeid 2023). Its complex genetics and antigenic diversity make it a captivating subject of study, attracting the attention of scientists, virologists, and immunologists worldwide.

IBV, formally designated as the species *Gammacoronavirus galli*, belongs to the *Gammacoronavirus* genus of the *Coronaviridae* family. The adaptability of specific IBV lineages is largely influenced by the evolutionary benefits of mutations in their structural genes, which help them adjust to evolving environmental pressures. IBV genomes undergo various genetic alterations, such as point mutations, deletions, insertions, and recombination, which contribute to the development of new virus variants, especially impacting structural genes like the S gene. For instance, IBV strains isolated from vaccinated birds show a mutation rate of 1.5% per year in the hypervariable regions of the S1 gene. In contrast, in the absence of vaccine-induced immunity, IBV evolves more slowly, at a rate of 0.3% annually in these same regions (Lee and Jackwood 2001). Alongside natural immunity and environmental influences, antibodies produced by IBV vaccines create selection pressure that accelerates genetic variation in the virus. This rapid evolution results in the development of new genotypes and serotypes.

The S1 gene is a critical focus in IBV research due to its pivotal role in determining the antigenic diversity of IBV strains. This gene encodes the spike glycoprotein, a key determinant of viral tropism and pathogenesis (Kuo et al. 2000). The S1 gene’s high genetic variability, driven by selection pressures such as host immune responses and interspecies transmission, is indicative of the virus’s dynamic evolution. Consequently, the S1 gene is invaluable for phylogenetic analyses to reconstruct evolutionary histories, track lineages, and identify emerging variants. Comprehensive comparative studies enabled by extensive S1 gene sequence databases offer critical insights into IBV epidemiology and the distribution of genetic diversity across geographic regions and poultry populations. Currently, IBV strains are classified into 36 lineages within 7 distinct genotypes (GI–GVII) (Valastro et al. 2016) based on phylogenetic analysis of the S1 gene sequence using maximum likelihood techniques (Felsenstein 1981).

Past studies have used phylogenetic trees to uncover the evolutionary lineage of IBV strains, enabling researchers to infer their history and pinpoint distinct lineages and potential sources of new outbreaks. However, analyzing IBV strains through phylogenetic methods faces challenges including the virus’s rapid mutation rate, potential recombination altering genetic relationships, and the necessity for comprehensive and representative sequence data to ensure precise tree construction. In larger systems, character-based tree construction also encounters issues related to consistency, efficiency, and robustness. On the other hand, phylogenetic networks provide a flexible method that can handle reticulate events and reconcile conflicting signals in the data (Huson et al. 2010). These networks are graphical representations used in evolutionary biology to illustrate the intricate relationships among genetic sequences. They provide a more nuanced depiction of evolutionary history, particularly in cases where traditional tree structures might oversimplify relationships. Recent progress includes the development of algorithms and tools designed to ensure the efficient, consistent, and reliable construction of these networks (Hu et al. 2017; Ge et al. 2022). Phylogenomic networks, which integrate information from multiple phylogenetic networks, are a powerful tool for generating comprehensive evolutionary insights (Hu et al. 2023).

By employing a distance-based method for constructing phylogenetic networks, this study examines the influence of migratory birds on the clustering, evolutionary patterns, and recombination events of IBV strains. Leveraging the conserved S1 gene sequence data, the method offers an efficient, robust, and accurate alternative for elucidating IBV evolutionary relationships. By reconstructing the evolutionary history of IBV strains within a network framework, this approach provides valuable insights into the virus’s diversification and geographical spread. Additionally, the network structure facilitates the direct analysis of recombination events and selection pressures acting on distinct viral subpopulations.

## 2. Materials and methods

### 2.1. IBV dataset

To create a reliable and effective phylogenetic network of IBV for studying its classification and evolution, we utilized a dataset of 311 IBV strains (see Table S1 in the supporting information). The dataset comprises 199 sequences derived from a representative subset of Valastro et al. (2016) and an additional 112 sequences obtained from the NCBI GenBank database to expand geographic coverage. Samples were collected from various geographical locations and over several years, providing a comprehensive dataset for analysis. Given the closely related nature of these IBV strains within the coronavirus family, we focused on their conserved S1 gene to construct the phylogenetic network.

### 2.2. Distance matrix of IBV

The IBV distance matrix is essential in phylogenetics for quantifying genetic differences between IBV strain sequences. We compute the evolutionary distance among IBV strains through the following steps:

1. Aligning their S1 gene using MAFFT (a multiple sequence alignment tool) version 7 (Katoh and Standley 2013).
2. Employing IQ-TREE 2 (a phylogenetic analysis tool) to identify the optimal model for S1 gene evolution (Minh et al. 2020).
3. Using Phangorn (a phylogenetic analysis tool) to calculate the distance matrix based on the identified optimal model for our dataset (Schliep 2011).

MAFFT is widely respected in bioinformatics for its rapid and accurate sequence alignment, which is critical across various biological studies. IQ-TREE 2 is a widely recognized software package for phylogenetic analysis known for its efficiency in handling large datasets. Through maximum-likelihood estimation, IQ-TREE 2 aids in identifying the most suitable model for IBV evolution, ensuring an accurate assessment of their evolutionary relationships. Detailed results of the character-based phylogenetic analysis, including BIC, AIC, and AICc (which corrects for small sample sizes to prevent overfitting) scores, are provided in Table S2 of the supporting information. Based on these scores, TIM3 + F + R10 emerges as the most suitable evolution model for the 311 IBVs studied (Posada 2003). This model incorporates three key components that make it particularly suitable for viral sequence analysis: the transitional model (TIM3) accounts for the higher likelihood of transitions versus transversions in nucleotide substitutions, which is especially relevant for RNA viruses like IBV where transition mutations are more frequent; the unequal base frequencies (+F) parameter acknowledges the known nucleotide composition bias in viral genomes, particularly in IBV where specific nucleotide frequencies may be influenced by host adaptation and replication mechanisms; and the rate heterogeneity model with 10 categories (+R10) captures the varying evolutionary rates across different genome positions, crucial for viral genes that experience different selective pressures. While simpler models might underfit the data by ignoring these biological realities, and more complex models could potentially overfit, the TIM3 + F + R10 model strikes an optimal balance by incorporating key evolutionary features while maintaining statistical tractability. Additionally, Phangorn is a highly regarded R package known for its reliability and computational efficiency. It excels in calculating evolutionary distances between genes, which is essential for phylogenetic analysis. Its versatility in handling a wide range of evolutionary models makes it an invaluable tool for examining genetic relationships and evolutionary dynamics across individual genes or entire genomes. Detailed information about the tools and software is available in the supporting information.

### 2.3. MSClustering method

**Figure 1.**
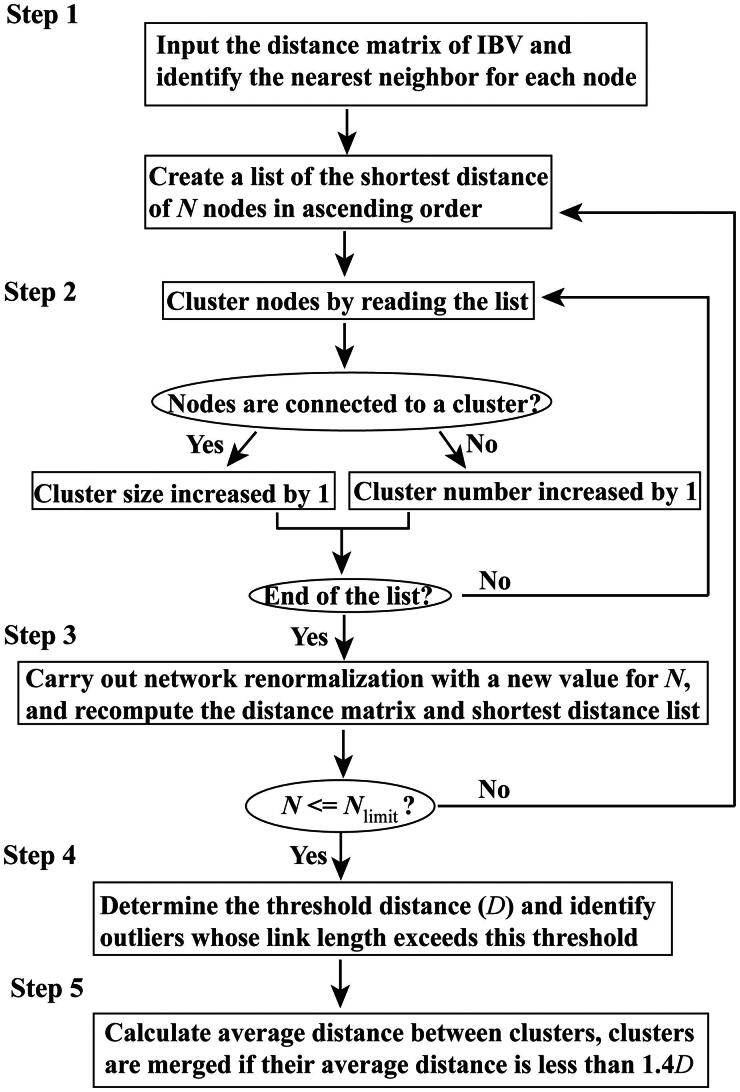
Flowchart outlining the steps of the revised MSClustering method used to build phylogenetic networks. The method considers the total number of nodes (*N*) and aims to produce a specified number of clusters (*N*_limit_, default 1).

In this study, we revised the MSClustering method to analyze, classify, and visualize the phylogenetic network of IBV using its distance matrix (Hu et al. 2017; Ge et al. 2022). This method constructs the network by minimizing both the average distance within each cluster and the overall connected distance of the network. The revised MSClustering methodology employs a hierarchical approach to cluster and visualize the complex network structure across various levels of resolution, depicted in Figure 1. This subsection provides a concise overview of the importance of each step in the revised MSClustering procedure:

Step 1. Simplification: This step streamlines the clustering process by managing a sorted list of the *N* shortest distances, rather than handling a distance matrix with *N*^2^ elements.
Step 2. Clustering: In this step, the list of *N* distances from Step 1 is used to efficiently identify all clusters.
Step 3. Renormalization: This step involves classifying the IBV network at different characteristic levels of resolution.
Step 4. Outlier Detection: This step identifies outliers caused by limited system size, thereby improving accuracy and coherence.
Step 5. Cluster Merging: This step improves the coherence of the predicted clusters while preserving the consistent network topology across various scales.

The hierarchical clustering approach employed in the revised MSClustering method offers a flexible framework for exploring viral evolution at multiple levels of resolution. By varying the clustering resolution, researchers can uncover both broad evolutionary patterns and fine-grained genetic details. At a coarser level, the network can reveal major viral lineages and population structure, while at a finer level, it can highlight subtle genetic variations that may influence viral adaptation and pathogenicity. However, it’s crucial to recognize the trade-off between resolution and complexity. Increasing resolution can enhance the detection of subtle genetic differences but may lead to a more complex network, making interpretation challenging. Conversely, decreasing resolution simplifies the network but can obscure important genetic diversity. Therefore, striking a balance between resolution and complexity is essential to obtain a comprehensive and interpretable understanding of viral evolution. Detailed information on the specific parameters of MSClustering can be found in the supporting information.

While the MSClustering method offers significant advantages in terms of computational efficiency and scalability, it does have certain limitations that need to be considered. One limitation is the simplification step, where the clustering process is based on a sorted list of the *N* shortest distances rather than the full distance matrix. This approach reduces computational complexity but may sacrifice some accuracy, particularly in cases where the relationships between strains are less clear or where there are many closely related sequences. Additionally, while renormalization helps classify the IBV network at different levels of resolution, the threshold selection for determining resolution may not always be optimal for every dataset, potentially affecting the granularity of clustering. The outlier detection step aims to improve accuracy by identifying strains that deviate from the norm, but its effectiveness is limited by the size and diversity of the dataset, and may not always capture subtle but important evolutionary variations. Furthermore, cluster merging seeks to enhance the coherence of the predicted clusters, yet this step can sometimes lead to over-simplification, particularly when there are more complex, heterogeneous patterns of viral evolution. Finally, while the method provides substantial improvements in processing speed, the statistical limitations associated with threshold estimation for smaller networks could affect the precision of the clustering results. Future refinements to these steps, particularly in terms of parameter optimization and the handling of complex datasets, could further improve the accuracy and robustness of the MSClustering method.

### 2.4. Selection pressure estimation

In genetics, the Nei-Gojobori (NG) method is a widely used statistical technique in molecular evolution studies to assess evolutionary pressures on a gene (Nei and Gojobori 1986). It does so by comparing the rates of amino acid-altering mutations (non-synonymous, dN) with silent mutations (synonymous, dS). The dN/dS ratio, which represents the proportion of non-synonymous to synonymous substitution rates, serves as an indicator of selection pressures. A ratio around 1 suggests neutral evolution (ln(dN/dS) = 0), a ratio less than 1 indicates negative selection (ln(dN/dS) < 0), and a ratio greater than 1 points to positive selection (ln(dN/dS) > 0) affecting a group of homologous protein-coding genes. Given the moderate divergence of the S1 gene in IBV strains within our dataset, we employ the NG method to estimate selection pressures.

Despite its widespread use, the NG method has several limitations. One major limitation is its sensitivity to sequence alignment quality; misalignments can distort dN and dS estimates, leading to inaccurate conclusions about selection pressures. The method also assumes a constant rate of evolution across all sites, which may not account for variable mutation rates and selection pressures within different gene regions, potentially skewing results. Additionally, the NG method does not consider complex evolutionary dynamics such as recombination or gene conversion, which can impact substitution rates and accuracy. Finally, its reliance on a simplistic nucleotide substitution model can be limiting, particularly in systems with diverse mutation types and varying substitution rates. Therefore, while the NG method provides useful approximations, it should be complemented with additional analyses and models for a more accurate estimation of selection pressures in complex systems.

To address these limitations, more sophisticated models, such as the Fixed Effects Likelihood (FEL) model (Kosakovsky Pond and Frost 2005), offer a more nuanced approach. The FEL model accounts for site-specific variation in selection pressures across the entire phylogeny, enabling a more precise estimation of positive and negative selection at individual codon sites. This allows for the detection of subtle patterns of selection that may be overlooked by simpler methods, providing valuable insights into the molecular mechanisms underlying adaptation and disease evolution. As such, complementing the NG method with advanced models like FEL can improve the accuracy and depth of selection pressure analyses in complex genetic systems.

### 2.5. Recombination event analysis

Recombination is crucial for IBV evolution, generating diverse variants by exchanging genetic material between strains. This process impacts viral adaptation, pathogenicity, and the emergence of new strains. Understanding recombination patterns aids in vaccine development, outbreak tracking, and predicting future viral trends, ultimately improving poultry health management.

We employed RDP5 (Recombination Detection Program version 5) to identify and analyze recombination events within aligned nucleotide sequence data (Martin et al. 2021). RDP5 was selected for its comprehensive toolset, which integrates multiple methods and advanced algorithms, coupled with a user-friendly interface. This software is renowned for its high accuracy and reliability in detecting recombination events, making it an invaluable resource for studying viral evolution. Upon providing a dataset of 311 IBV sequences, RDP5 utilizes various methods to detect regions of similarity between query sequences and reference sequences within the dataset. It then applies statistical tests to determine if the observed patterns deviate from simple inheritance patterns, thus indicating potential recombination events. RDP5 offers diverse outputs, including the statistical significance of detected recombination events, breakpoint locations, and graphical representations of the findings. Our analysis focuses on potential recombination events identified using a stringent *P*-value threshold of 5 × 10^−4^ for seven detection methods: RDP, GENECONV, Bootscan, Maxchi, Chimaera, SiSscan, and 3Seq. This threshold is notably smaller than the 0.05 required to meet the test’s criteria.

## 3. Results

### 3.1. Phylogenetic network of IBV

**Figure 2.**
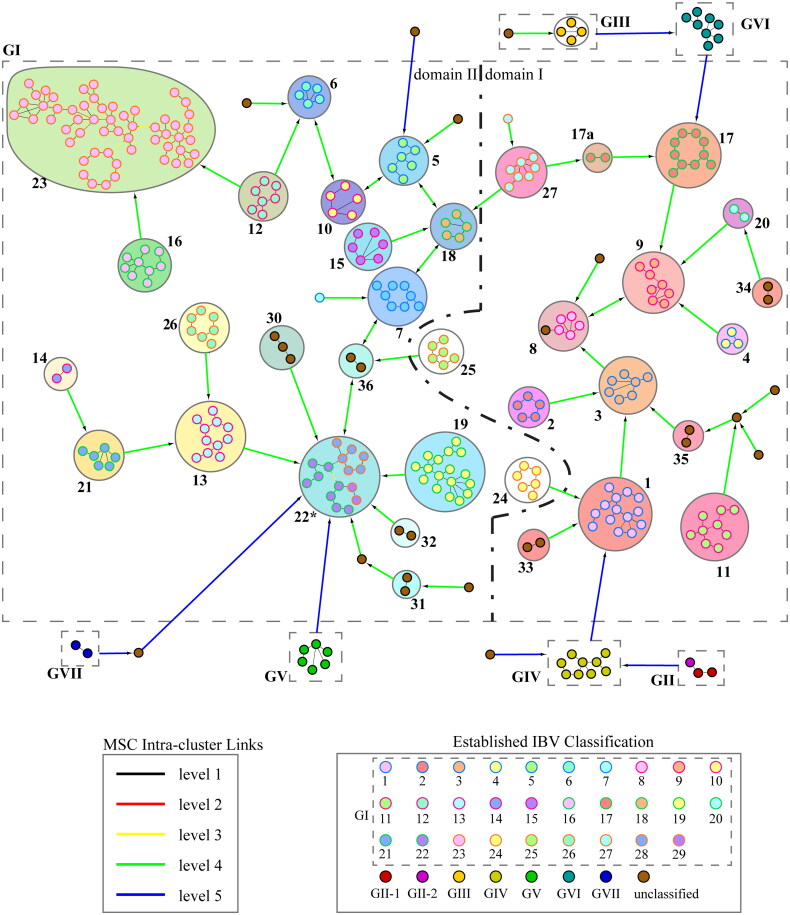
Phylogenetic network diagram depicting 311 IBV strains, with each strain represented as a node and color-coded based on its established classification. Brown nodes represent previously unclassified IBV strains. The edges connecting nodes are color-coded: black for level 1 intra-cluster links and blue for level 5 intra-cluster links. These edges are directed towards local hubs within the network. Genotypes are delineated by dashed lines, while lineages are delineated by solid lines. An isolated node signifies an outlier. The color coding for GI clusters within the solid line (excluding 24 and 25) represents both their network positions and the geographic regions where these strains are found. A detailed legend explaining the color scheme for each GI cluster is provided in Figure 7.

Figure 2 illustrates the phylogenetic network of 311 IBV strains derived from MSClustering, with nodes representing individual IBVs color-coded according to their established classification (Valastro et al. 2016; Chen et al. 2017; Jiang et al. 2017; Ma et al. 2019; Hou et al. 2020; Moharam et al. 2020; Houta et al. 2021). The network is organized into 7 genotypes (GI to GVII), with genotype GI further divided into 35 lineages. Detailed clustering results are available in Table S1 of the supporting information.

In line with existing research, we clustered IBVs at levels 3 and 4, using dashed and solid lines to visually differentiate these clusters. At level 4, the dataset is categorized into 7 genotypes and 3 outliers, consistent with established classifications. At level 3, genotype GI is further divided into 35 lineages and 10 outliers. Our analysis introduced a new group, GI-22*, by merging lineages 22, 28, and 29, based on smaller distances between GI-22 and GI-29 and GI-28 compared to the intra-cluster distances within GI-22. Additionally, lineage 17 was split into two clusters, GI-17 and 17a, due to a larger genetic distance between them. We note that MSClustering results may vary with different threshold values, particularly in small networks where statistical estimation has limitations.

Most GI lineages show geographic specificity, with many found mainly in certain regions. However, some lineages, like GI-23, are more widely distributed across multiple continents. Figure 2 reveals that the GI subnetwork is divided into two main geographic domains: domain I, covering the Americas, and domain II, including Europe, Asia, Africa, and Oceania. This geographic distribution indicates that strains in the Americas show unique evolutionary patterns compared to other continents, likely influenced by geographic isolation.

The MSClustering results provide valuable insights into IBV evolution and diversity by categorizing strains based on genetic similarity. This classification uncovers distinct evolutionary pathways influenced by factors such as geographic location, host adaptation, and immune pressure. Detailed analysis of these clusters could reveal patterns of viral spread, emergence of new variants, and potential outbreak hotspots, thereby offering a solid framework for understanding IBV dynamics and guiding disease control and prevention strategies.

### 3.2. Comparing phylogenetic trees of IBV

Phylogenetic trees are constructed using either distance-based or character-based methods. Distance methods group sequences based on overall genetic similarity, while character-based methods analyze individual character changes to infer relationships. Distance methods are computationally efficient but may overlook complex evolutionary patterns, whereas character-based methods provide more detailed information but are computationally demanding for large datasets.

**Figure 3.**
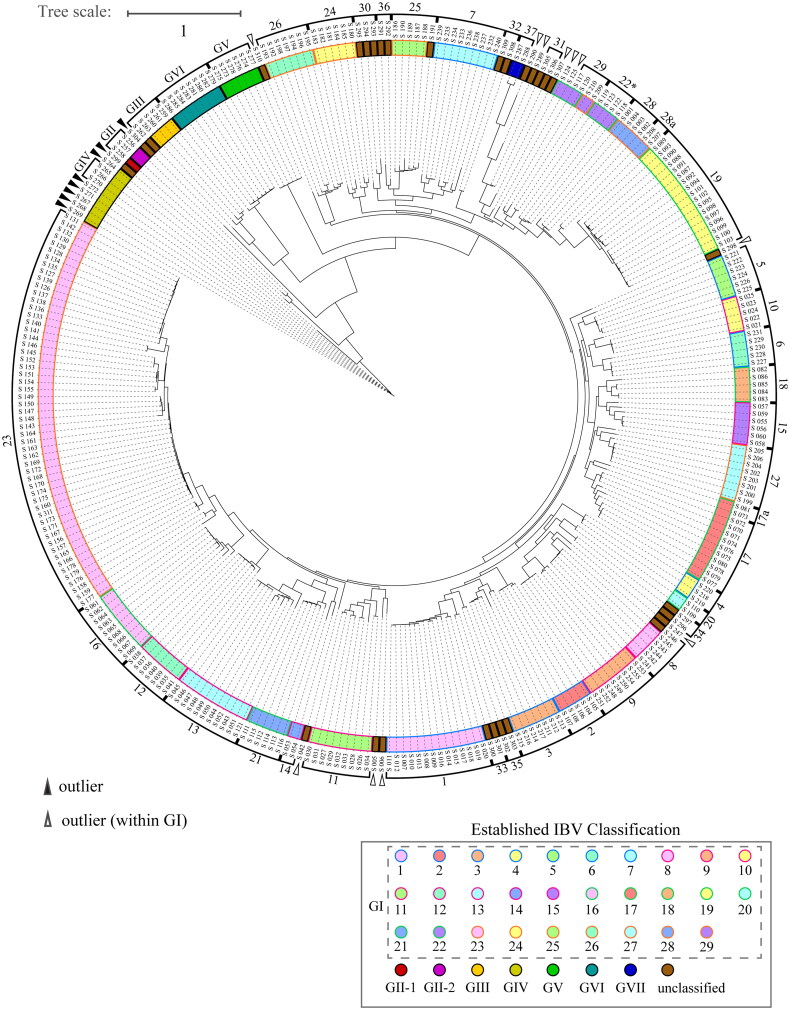
Phylogenetic tree of 311 IBV strains constructed using the GTR model with PhyML. IBV strains are color-coded according to established IBV classifications in the inner circle, with manual classifications based on this predicted tree shown on the outer rim. The color coding scheme for the strains is consistent with Figure 2. Brown nodes represent unclassified IBV strains.

Figure 3 presents a character-based phylogenetic tree of the IBV strains analyzed using PhyML 3.0 with the GTR substitution model and 100 bootstrap replicates for branch support (Guindon et al. 2010). PhyML was selected for comparison because it was used to construct the established classification for a slightly different dataset (Valastro et al. 2016). This analysis categorized 311 strains into 6 genotypes, with genotype GI further divided into 36 lineages. Detailed results are provided in Table S3 of the supporting information. Overall, the classifications are consistent, showing minor discrepancies: 7.1% between PhyML and MSClustering, 5.5% between PhyML and the established classification, and 3.9% between MSClustering and the established classification. Inconsistencies are noted in lineages 22, 28, and 29, with PhyML reclassifying some strains from GVII into GI and strains from GIV as outliers, likely due to the limitations of the heuristic search method used.

The discrepancies between MSClustering and traditional character-based methods like PhyML fundamentally stem from their distinct analytical frameworks and assumptions. Character-based methods rely on shared sequence characteristics to reconstruct evolutionary relationships through phylogenetic trees, using principles like maximum likelihood to identify the most probable evolutionary scenario. While this approach excels at tracking specific sequence changes, it assumes a strictly tree-like evolution that may oversimplify the complex reality of viral evolution, particularly for viruses like IBV that frequently undergo recombination. In contrast, MSClustering employs a network-based approach that identifies clusters of related sequences without enforcing a rigid tree structure, making it better suited for capturing complex evolutionary events like horizontal gene transfer and recombination. Moreover, while character-based methods can be sensitive to sequence noise and alignment errors, MSClustering demonstrates greater robustness by focusing on broader genetic similarity patterns rather than individual nucleotide changes. These methodological differences suggest that combining both approaches provides a more nuanced understanding of IBV evolution—traditional methods excel at detailing specific evolutionary changes, while MSClustering better captures the complex network of relationships that characterize viral adaptation and diversity.

We evaluated the performance of our methods on an Intel^®^ Core^™^ i9-11900F @ 2.50 GHz CPU with 8 cores. The MSClustering method processed the entire dataset in just 1.4 s, demonstrating exceptional efficiency. In contrast, PhyML required a significantly longer time (1.3 × 10^5^ s) to find the consensus phylogenetic tree with 100 bootstrap replicates. This substantial difference highlights the computational advantage of the MSClustering approach for large datasets.

### 3.3. Evolution of IBV’s S1 gene

**Figure 4.**
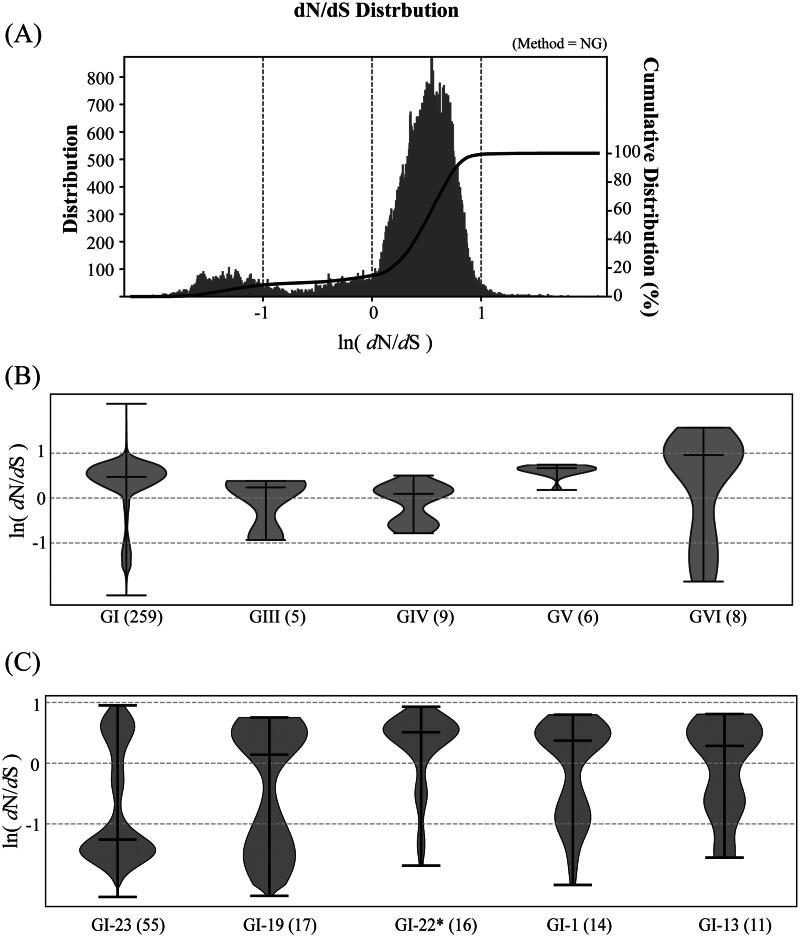
Estimation of selection pressure for the 311 IBV dataset. (A) Distribution and cumulative distribution of ln(dN/dS) for the entire dataset. Negative ln(dN/dS) values denote negative selection, while positive values indicate positive selection. (B) Violin plots of ln(dN/dS) for genotypes GI and GIII-GVI. (C) Violin plots of ln(dN/dS) for the largest five lineages in GI. In (B) and (C), the three lines from top to bottom indicate the maximum, median, and minimum values of the data. The numbers in parentheses indicate the number of IBV strains in each cluster.

Figure 4 illustrates the distribution of ln(dN/dS) values for the S1 gene of IBV strains calculated using the NG method. It presents the results for the entire dataset (A), intra-genotype pairs (B), and intra-lineage pairs within GI (C). In panel (A), the distribution shows two peaks: the first peak in the distribution centers at −1.33, indicating negative selection, while the second peak centers at 0.55, suggesting positive selection. The cumulative distribution shows that 84.6% of the entire dataset exhibits positive selection, indicating substantial selection pressure in the evolution of IBV.

In Figure 4(B), we depict the violin plot of ln(dN/dS) values for genotypes GI and GIII-GVI. The width of each violin represents the distribution of data points within that group, with a wider section indicating a higher distribution. GII and GVII are excluded due to their small group size. As GI contains 83% of the 311 IBV strains, its distribution closely resembles that in (A). Across different genotypes, the average value of ln(dN/dS) varies. It is 0.39 for GI, 0.07 for GIII, −0.04 for GIV, 0.62 for GV, and 0.73 for GVI. It is evident that genotypes GV and GVI are less conserved due to selection pressure and GIV is the most conserved. Figure 4(C) showcases the violin plot of ln(dN/dS) values for the five largest lineages within GI. The strains in the largest lineage, GI-23, exhibit significantly higher conservation compared to the other four lineages. Strains within GI-22* are mainly found in China, whereas those in the remaining lineages are distributed across multiple countries. Intriguingly, our analysis indicates that GI-22* undergoes a higher degree of diversifying selection compared to the other lineages.

**Figure 5.**
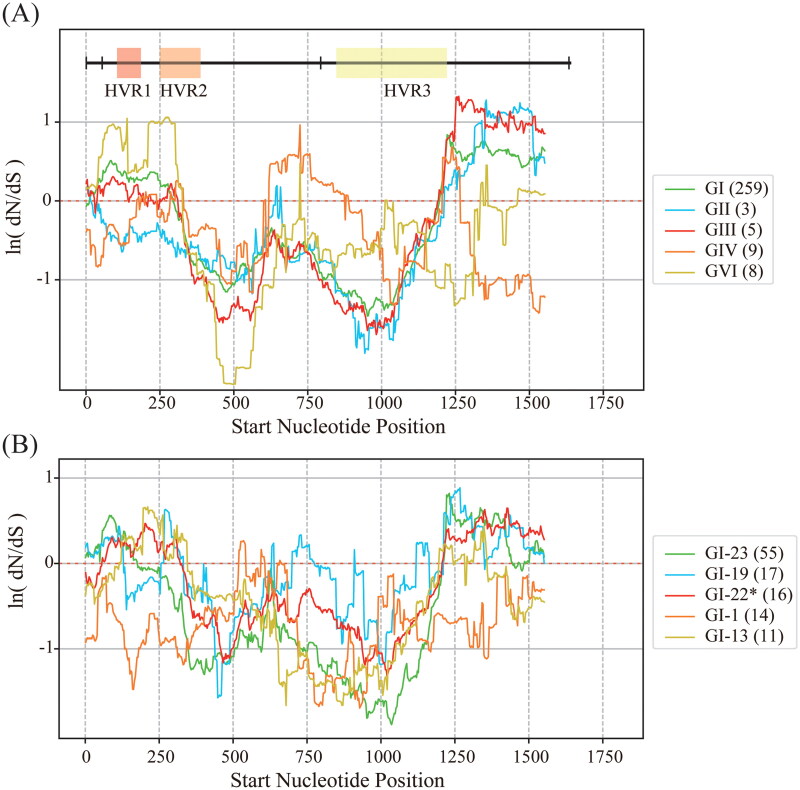
Sliding window analysis of ln(dN/dS) values along the S1 sequences of IBV strains for different genotypes (GI-GIV and GVI) in (A) and various lineages (1–5) within GI in (B). The window size is 90 nucleotides with a step size of 3 nucleotides. Approximate ranges of HVR1, HVR2, and HVR3 in the S1 gene are displayed, which may vary for different strains. Negative ln(dN/dS) values denote negative selection, while positive values indicate positive selection. Numbers in parentheses indicate the number of IBV strains in each cluster.

To systematically explore the evolution of hypervariable regions (HVRs) in the receptor-binding subunit S1, we computed the average dN/dS ratio across a 90-nucleotide sliding window along its nucleotide sequence for various genotypes and lineages of IBV. The S1 gene typically encompasses three HVRs roughly located within nucleotides 112–201 (HVR1), 271–423 (HVR2), and 820–1161 (HVR3), although their positions may vary slightly among different strains (Abdel-Moneim et al. 2006). Figure 5 illustrates the average ln(dN/dS) values for genotypes GI, GII-GIV, and GVI (A), and for the largest five lineages 23, 19, 22*, 1 and 13 within GI (B), indicating that most IBV strains experience significant selection pressure in the front (nucleotides 1–300), middle (nucleotides 630–885), and rear (nucleotides 1240–) regions. In panel (A), genotypes GI, GIII, and GVI exhibit substantial selection pressure within all three HVR regions, while genotypes GII and GIV display significant pressure primarily within HVR3. Given that most IBV strains belong to GI, panel (B) further examines the selection pressure within the largest five lineages of GI. Generally, lineages 19, 22*, and 13 show notable selection pressure across all three HVR regions, whereas lineage 23 demonstrates variability in HVR1 and HVR3. Notably, lineage 1’s S1 gene remains highly conserved, except for nucleotides around 540.

To summarize our findings on IBV evolution under selection pressure, we focus on strains experiencing intense selection pressure, specifically genotype GVI and lineage 22* of GI, all of which are found in China or Korea. These strains, influenced by environmental factors such as host immune responses or drug treatments, can rapidly acquire genetic changes that enhance their transmissibility, virulence, or drug resistance. Monitoring these strains is essential for predicting outbreaks, developing effective countermeasures, and understanding the mechanisms of viral adaptation. Staying ahead of these evolving pathogens is crucial for protecting public health and mitigating the impact of future outbreaks.

### 3.4. Recombination events of IBVs

**Figure 6.**
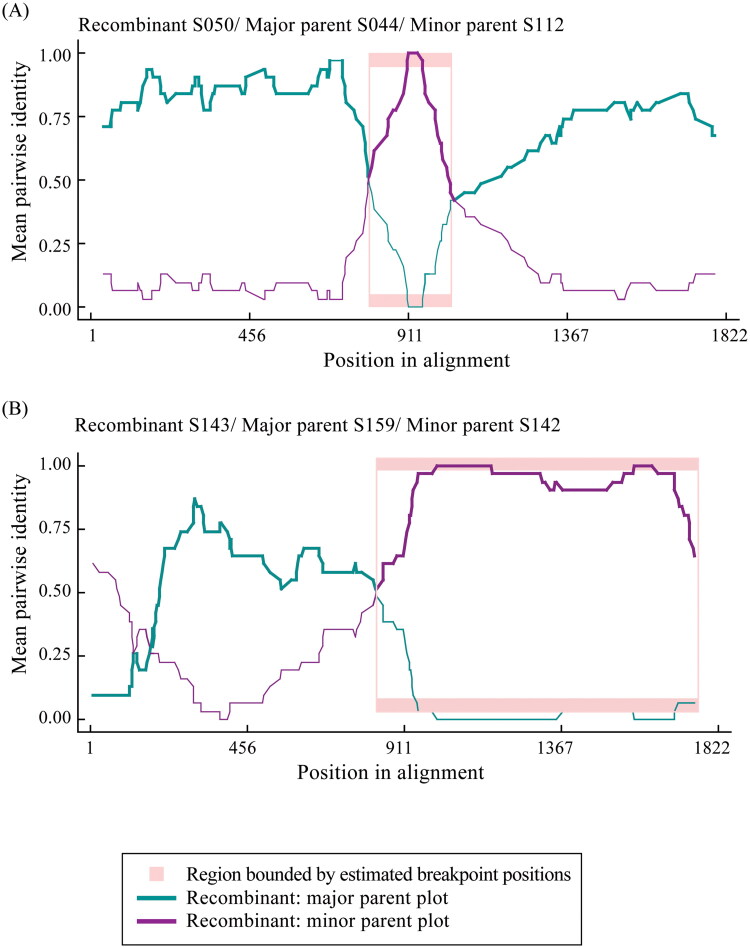
Pairwise identity plots of potential recombination events: (A) Recombinant S050, major parent S044, and minor parent S112, and (B) Recombinant S143, major parent S159, and minor parent S142. The thicker line represents the pairwise identity of the recombinant sequence compared to its parental sequences. The pink region between breakpoints indicates a segment of the recombinant sequence that exhibits high similarity to the minor parent, suggesting a recombination event.

Our network improves the detection of non-treelike evolutionary patterns, such as recombination. Table S4 in the supporting information summarizes potential recombination events in the S1 gene of 311 IBVs. Notably, we focus on two recombination events, S050 and S143, which satisfy the criterion of occurring after their parent sequences. Figure 6 shows their pairwise identity plots: (A) Recombinant S050, with breakpoints at nucleotides 802 and 1016, is more similar to its major parent S044 than to the minor parent S112. (B) Recombinant S143, with breakpoints at nucleotides 856 and 1792, is a roughly equal mix of its major parent S142 and minor parent S158.

Identifying recombination events in IBV is essential for monitoring the virus’s evolution, as such events can give rise to new strains with altered virulence or vaccine resistance. Recombination occurs when two distinct viral strains infect the same host cell and exchange genetic material, creating hybrid viruses with combined traits from both strains. These recombinant strains may carry mutations in genes that code for key viral proteins, such as the spike protein, which is vital for viral entry into host cells and evading immune detection. Changes to the spike protein or other surface proteins can reduce the virus’s susceptibility to neutralizing antibodies from previous infections or vaccinations, weakening the effectiveness of existing vaccines. This can lead to the emergence of vaccine-resistant strains, complicating efforts to control outbreaks. Additionally, recombination can introduce genetic elements that increase the virus’s ability to cause disease, enhance replication, or bypass host immune defenses. For example, a recombined strain may inherit virulence factors from one parent strain while acquiring immune evasion strategies from another, potentially resulting in a more severe or persistent infection. Understanding these dynamics is critical for developing more effective vaccines, improving epidemiological surveillance, and strengthening biosecurity measures. By closely monitoring these genetic changes, researchers and health authorities can respond more quickly to new strains, helping to protect poultry health and reduce the economic impact of outbreaks.

## 4. Discussion

IBV-induced infectious bronchitis, a respiratory disease in chickens, emerged in the early 1930s and has become a widespread problem for intensive poultry operations around the globe (Jackwood and de Wit 2013). The epidemiology of IBV is multifaceted, influenced by factors such as poultry trade, biosecurity practices, and the role of migratory birds. Migratory birds have been identified as natural hosts of IBV (Wille and Holmes 2020). Studies have documented the presence of IBV strains in various wild bird populations (Hughes et al. 2009). Furthermore, the potential for viral evolution at the agriculture-wildlife interface, where domestic poultry flocks and migratory bird habitats overlap, cannot be ignored (Ayala et al. 2020). While poultry trade undoubtedly poses a significant risk for the introduction and spread of IBV, migratory birds emerge as another crucial factor in understanding IBV epidemiology.

Unlike trade-introduced strains, which typically exhibit significant genetic divergence from local strains, our phylogenetic network reveals a distinct pattern for many IBV lineages. These lineages often display close genetic similarity and a geographically clustered distribution. This observation suggests a transmission pathway beyond established poultry trade routes. For instance, the GI-1 lineage originated in the U.S. and has spread globally to Europe, Asia, and Africa since the 1950s (Chen et al. 2023). Within the phylogenetic network, GI-1 is deliberately colored to emphasize its U.S. origin. This distinct coloring highlights the possibility of independent dissemination pathways for this strain, independent of established trade influences. This suggests that migratory birds may play a role in shaping the evolutionary relationships observed among IBV lineages within our phylogenetic framework.

**Figure 7.**
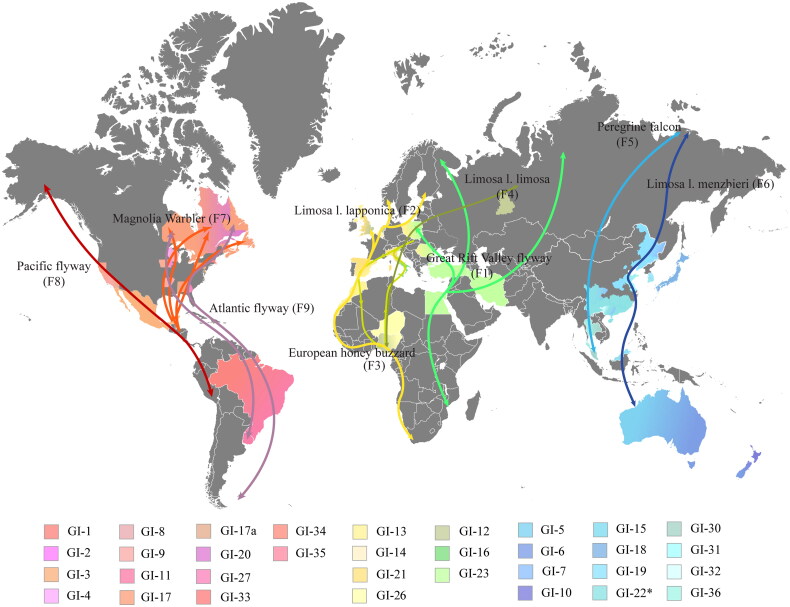
Global map illustrating bird migratory routes F1–F9 and the regions where IBV strains were identified. As indicated in the legend, regions are color-coded according to our predicted IBV lineages, reflecting both their network position and the geographic location. The consistent color scheme—red, yellow, green, and blue—correlates closely positioned GI lineages in both the phylogenetic network and geographical regions worldwide.

Figure 7 illustrates the global migratory flyways (F1–F9) of birds and their potential impact on the evolutionary dynamics depicted in Figure 2. Both figures employ color-coded markers to categorize our predicted clusters within the GI group, based on their network connections and geographical spread. The consistent color scheme—red, yellow, green, and blue—correlates closely positioned GI lineages in both the phylogenetic network and geographical regions worldwide. For instance, GI-23 is highlighted as a case study, with IBV strains identified in Poland, Romania, and the Middle East (depicted in green in Figure 7), potentially associated with the Great Rift Valley migratory flyway (F1). This migratory route begins in Israel, extends south through Egypt, northeast through Iran, north through Turkey, and northwest through Romania to Poland. This alignment is reflected in the configuration of lineage GI-23 shown in Figure S1, where its branches extend from Israel to North and Central Egypt on one side, and to Turkey, Iran, Romania, and Poland on the other. In another instance, similarities observed between flyways F9 and F7 and the network lineage path 26-35-3-8-9-17 within domain I underscore potential correlations between bird migratory routes and the geographic locations where related IBV strains have been identified. The correlation between migratory bird routes and the evolutionary patterns of IBV lineages in the network indicates a potential role of migratory birds in IBV epidemiology.

**Figure 8.**
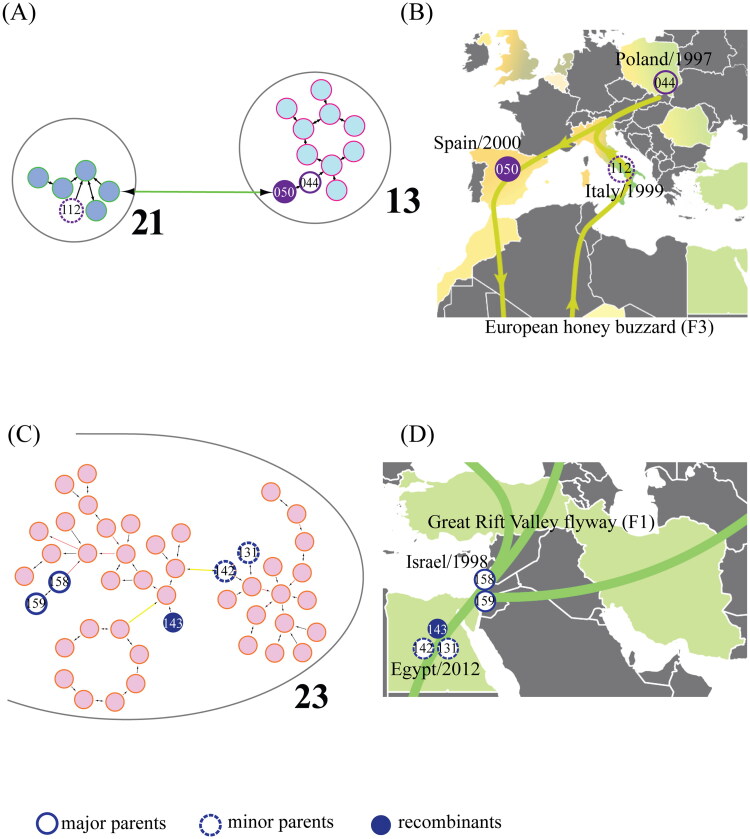
Identification of two potential recombination events within the IBV dataset: (A) partial network diagram indicating the position of recombinant sequence S050 and its parent sequences. (B) Geographical map displaying the bird migratory route F3 associated with recombinant sequence S050 and its parent sequences. (C) Partial network diagram indicating the position of recombinant sequence S143 and its parent sequences. (D) Geographical map displaying the bird migratory route F1 associated with recombinant sequence S143 and its parent sequences. Filled circles represent recombinants, open circles denote major parents, and open dashed circles denote minor parents, with the number in the circles indicating the ID of IBV strains.

Our study also investigated the potential role of migratory birds in IBV recombination events. As illustrated in Figure 8(A), the IBV strain S050 (Spain, 2000) appears to have emerged from a recombination event. Strain S044 (Poland, 1997) is identified as the major parent sequence, while S112 (Italy, 1999) acts as the minor parent. Given the minimal poultry export from Poland or Italy to Spain in 1999, this suggests a transmission pathway beyond poultry trade. Interestingly, as shown in Figure 8(B), all these countries (Spain, Poland, and Italy) lie within the European honey buzzard flyway (F3). This geographical overlap strengthens the possibility of migratory bird involvement in the recombination event. Similarly, Figure 8(C) depicts a potential recombination event for strain S143 (Egypt, 2012). Strains 158 and 159 (Israel, 1998) are presumed to be the major parent sequences, while S131 and S142 (Egypt, 2012) act as minor parents. Given the absence of poultry export from Israel to Egypt in 2012, the potential for this recombination to occur along the Great Rift Valley flyway (F1), as shown in Figure 8(D), aligns with the migratory patterns of birds in this region. Detailed poultry trade analysis is provided in Figure S2 of the supporting information. These analyses of recombination events, along with the geographical distribution of the involved strains, support the hypothesis that migratory birds play a significant role in the evolution of IBV.

Therefore, incorporating migratory bird patterns into IBV surveillance and control strategies becomes essential. This might involve monitoring wild bird populations near poultry farms, developing vaccines effective against strains carried by wild birds, and implementing stricter biosecurity measures at the agriculture-wildlife interface. Given the intense selection pressure on IBV strains in genotype GVI and lineage 22* of GI in China and Korea, heightened surveillance in these regions is warranted. By acknowledging the role of migratory birds in IBV epidemiology, we can develop more comprehensive control strategies to safeguard the health of our poultry flocks.

## 5. Conclusion

In conclusion, this study provides a novel approach for understanding the evolutionary dynamics and transmission patterns of IBV, an important pathogen in poultry. By introducing the MSClustering method, we demonstrated its efficiency in clustering IBV strains and revealing distinct phylogenetic relationships, offering a faster and more scalable alternative to traditional methods. Our findings, particularly the unrecognized role of migratory birds in IBV dissemination, suggest that the virus may be spread through transmission routes beyond the conventional poultry trade networks, which could have significant implications for control strategies. The integration of phylogenetic network analysis and recombination event analysis enhances our understanding of IBV evolution and provides insights into potential new avenues for intervention.

Moving forward, further research is warranted to corroborate the potential impact of migratory birds on IBV evolution and distribution. Incorporating ecological and epidemiological data on avian migration patterns, along with more comprehensive viral sampling across diverse geographical regions, could provide valuable insights. Continued refinement of phylogenetic methods, such as MSClustering, can improve the accuracy and resolution of analyses. Additionally, the application of the MSClustering method to other viral pathogens may yield similar efficiency gains and facilitate large-scale phylogenetic analyses that inform our understanding of disease transmission and control.

## Supplementary Material

Supplemental Material

## References

[CIT0001] Abdel-Moneim AS, El-Kady MF, Ladman BS, Gelb J. 2006. S1 gene sequence analysis of a nephropathogenic strain of avian infectious bronchitis virus in Egypt. Virol J. 3(1):78. doi: 10.1186/1743-422X-3-78. 16987422 PMC1592083

[CIT0002] Abozeid HH. 2023. Global emergence of infectious bronchitis virus variants: evolution, immunity, and vaccination challenges. Transboundary Emerging Dis. 2023(1): 1144924. doi: 10.1155/2023/1144924. PMC12017171 40303661

[CIT0003] Ayala AJ, Yabsley MJ, Hernandez SM. 2020. A review of pathogen transmission at the backyard chicken–wild bird interface. Front Vet Sci. 7:539925. doi: 10.3389/fvets.2020.539925. 33195512 PMC7541960

[CIT0004] Chen L, Jiang W, Wu W, Zhang S, Cai J, Lv T, Xiang B, Lin Q, Liao M, Ding C, et al. 2023. Insights into the epidemiology, phylodynamics, and evolutionary changes of lineage GI-7 infectious bronchitis virus. Transboundary Emerging Dis. 2023(1):1–13. doi: 10.1155/2023/9520616. PMC12016960 40303710

[CIT0005] Chen Y, Jiang L, Zhao W, Liu L, Zhao Y, Shao Y, Li H, Han Z, Liu S. 2017. Identification and molecular characterization of a novel serotype infectious bronchitis virus (GI-28) in China. Vet Microbiol. 198:108–115. doi: 10.1016/j.vetmic.2016.12.017. 28062000 PMC7117283

[CIT0006] Felsenstein J. 1981. Evolutionary trees from DNA sequences: a maximum likelihood approach. J Mol Evol. 17(6):368–376. doi: 10.1007/BF01734359. 7288891

[CIT0007] Ge B-K, Hu G-M, Chen R, Chen C-M. 2022. MSClustering: a cytoscape tool for multi-level clustering of biological networks. Int J Mol Sci. 23(22):14240. doi: 10.3390/ijms232214240. 36430723 PMC9699063

[CIT0008] Guindon S, Dufayard J-F, Lefort V, Anisimova M, Hordijk W, Gascuel O. 2010. New algorithms and methods to estimate maximum-likelihood phylogenies: assessing the performance of PhyML 3.0. Syst Biol. 59(3):307–321. doi: 10.1093/sysbio/syq010. 20525638

[CIT0009] Hou Y, Zhang L, Ren M, Han Z, Sun J, Zhao Y, Liu S. 2020. A highly pathogenic GI-19 lineage infectious bronchitis virus originated from multiple recombination events with broad tissue tropism. Virus Res. 285:198002. doi: 10.1016/j.virusres.2020.198002. 32380209 PMC7198173

[CIT0010] Houta MH, Hassan KE, El-Sawah AA, Elkady MF, Kilany WH, Ali A, Abdel-Moneim AS. 2021. The emergence, evolution and spread of infectious bronchitis virus genotype GI-23. Arch Virol. 166(1):9–26. doi: 10.1007/s00705-020-04920-z. 33416996 PMC7791962

[CIT0011] Hu G-M, Mai T-L, Chen C-M. 2017. Visualizing the GPCR network: classification and Evolution. Sci Rep. 7(1):15495. doi: 10.1038/s41598-017-15707-9. 29138525 PMC5686146

[CIT0012] Hu G-M, Tai Y-C, Chen C-M. 2023. Unraveling the evolutionary patterns and phylogenomics of coronaviruses: a consensus network approach. J Med Virol. 95(11):e29233. doi: 10.1002/jmv.29233. 38009694

[CIT0013] Hughes LA, Savage C, Naylor C, Bennett M, Chantrey J, Jones R. 2009. Genetically diverse coronaviruses in wild bird populations of Northern England. Emerg Infect Dis. 15(7):1091–1094. doi: 10.3201/eid1507.090067. 19624927 PMC2744231

[CIT0014] Huson DH, Rupp R, Scornavacca C. 2010. Phylogenetic networks: concepts, algorithms and applications. Cambridge: Cambridge University Press.

[CIT0015] Jackwood MW, de Wit S. 2013. Infectious bronchitis. In: Diseases of poultry. New York: Wiley; p. 139–159.

[CIT0016] Jiang L, Zhao W, Han Z, Chen Y, Zhao Y, Sun J, Li H, Shao Y, Liu L, Liu S, et al. 2017. Genome characterization, antigenicity and pathogenicity of a novel infectious bronchitis virus type isolated from south China. Infect Genet Evol. 54:437–446. doi: 10.1016/j.meegid.2017.08.006. 28800976 PMC7106192

[CIT0017] Katoh K, Standley DM. 2013. MAFFT multiple sequence alignment software version 7: improvements in performance and usability. Mol Biol Evol. 30(4):772–780. doi: 10.1093/molbev/mst010. 23329690 PMC3603318

[CIT0018] Kosakovsky Pond SL, Frost SDW. 2005. Not so different after all: a comparison of methods for detecting amino acid sites under selection. Mol Biol Evol. 22(5):1208–1222. doi: 10.1093/molbev/msi105. 15703242

[CIT0019] Kuo L, Godeke GJ, Raamsman MJ, Masters PS, Rottier PJ. 2000. Retargeting of coronavirus by substitution of the spike glycoprotein ectodomain: crossing the host cell species barrier. J Virol. 74(3):1393–1406. doi: 10.1128/jvi.74.3.1393-1406.2000. 10627550 PMC111474

[CIT0020] Lee C-W, Jackwood MW. 2001. Origin and evolution of Georgia 98 (GA98), a new serotype of avian infectious bronchitis virus. Virus Res. 80(1–2):33–39. doi: 10.1016/s0168-1702(01)00345-8. 11597746

[CIT0021] Ma T, Xu L, Ren M, Shen J, Han Z, Sun J, Zhao Y, Liu S. 2019. Novel genotype of infectious bronchitis virus isolated in China. Vet Microbiol. 230:178–186. doi: 10.1016/j.vetmic.2019.01.020. 30827386 PMC7117389

[CIT0022] Martin DP, Varsani A, Roumagnac P, Botha G, Maslamoney S, Schwab T, Kelz Z, Kumar V, Murrell B. 2021. RDP5: a computer program for analyzing recombination in, and removing signals of recombination from, nucleotide sequence datasets. Virus Evol. 7(1):veaa087. doi: 10.1093/ve/veaa087. 33936774 PMC8062008

[CIT0023] Minh BQ, Schmidt HA, Chernomor O, Schrempf D, Woodhams MD, von Haeseler A, Lanfear R. 2020. IQ-TREE 2: new models and efficient methods for phylogenetic inference in the genomic era. Mol Biol Evol. 37(5):1530–1534. doi: 10.1093/molbev/msaa015. 32011700 PMC7182206

[CIT0024] Moharam I, Sultan H, Hassan K, Ibrahim M, Shany S, Shehata AA, Abo-ElKhair M, Pfaff F, Höper D, El Kady M, et al. 2020. Emerging infectious bronchitis virus (IBV) in Egypt: evidence for an evolutionary advantage of a new S1 variant with a unique gene 3ab constellation. Infect Genet Evol. 85:104433. doi: 10.1016/j.meegid.2020.104433. 32622080 PMC7327463

[CIT0025] Nei M, Gojobori T. 1986. Simple methods for estimating the numbers of synonymous and nonsynonymous nucleotide substitutions. Mol Biol Evol. 3(5):418–426. doi: 10.1093/oxfordjournals.molbev.a040410. 3444411

[CIT0026] Posada D. 2003. Using MODELTEST and PAUP* to select a model of nucleotide substitution. Curr Protoc Bioinformatics. Chapter 6(1):Unit 6.5. doi: 10.1002/0471250953.bi0605s00. 18428705

[CIT0027] Schliep KP. 2011. phangorn: phylogenetic analysis in R. Bioinformatics. 27(4):592–593. doi: 10.1093/bioinformatics/btq706. 21169378 PMC3035803

[CIT0028] Valastro V, Holmes EC, Britton P, Fusaro A, Jackwood MW, Cattoli G, Monne I. 2016. S1 gene-based phylogeny of infectious bronchitis virus: an attempt to harmonize virus classification. Infect Genet Evol. 39:349–364. doi: 10.1016/j.meegid.2016.02.015. 26883378 PMC7172980

[CIT0029] Wille M, Holmes EC. 2020. Wild birds as reservoirs for diverse and abundant gamma- and deltacoronaviruses. FEMS Microbiol Rev. 44(5):631–644. doi: 10.1093/femsre/fuaa026. 32672814 PMC7454673

[CIT0030] World Bank and TAFS Forum. 2011. World livestock disease atlas: a quantitative analysis of Global Animal Health Data (2006-2009). Washington, DC: World Bank.

