# Supplementary material for: Phylogenetic network of infectious bronchitis virus: exploring the impact of migratory birds on viral clustering, evolution, and recombination
Source: Vet Q. 2025 Feb 14;45(1):1–14. doi: 10.1080/01652176.2025.2465570 (PMC11834806; doi:10.1080/01652176.2025.2465570)
Supplement: Supplemental Material [file TVEQ_A_2465570_SM0905.zip › Suppl/supp_ibv3R3.pdf]

Supporting Information

For “Phylogenetic Network of Infectious Bronchitis Virus: Clustering, Evolution, and Recombination”
By Yu-Chen Tai, Geng-Ming Hu, and Chi-Ming Chen

Table of Contents

1. Supplementary Information for Methods (page 1).
2. Supplementary Comparison of IBV’s Phylogenetic Trees (page 2-3)
3. Supplementary Tables S1-S4 (pages 4-20).
4. Supplementary Figures S1-S2 (pages 21-22).

1. Supplementary Information for Methods

MAFFT : In our study, we employed parameters “--auto --inputorder” to enable MAFFT to automatically select the optimal alignment strategy based on dataset size and maintain the input sequence order in the alignment. This approach ensures alignment consistency and enhances the effectiveness of subsequent analyses.
IQ-TREE 2: AIC and BIC are information criteria that weigh model fit against complexity, preferring models that effectively explain data while penalizing excessive complexity. In IQ-TREE 2, selection of the model with the lowest score indicates a favorable balance between data fit and model complexity.
MSClustering: When constructing the phylogenetic network of IBV, we employed a threshold distance of 0.32 at level 4 and 0.14 at level 3. For merging clusters within GI at level 3, the threshold for the average cluster distance was set at 0.19. To assess the robustness of the MSClustering results, we calculated the Jaccard similarity between the clustering obtained and those generated using threshold values deviating from 0.19, as shown in the figure below.

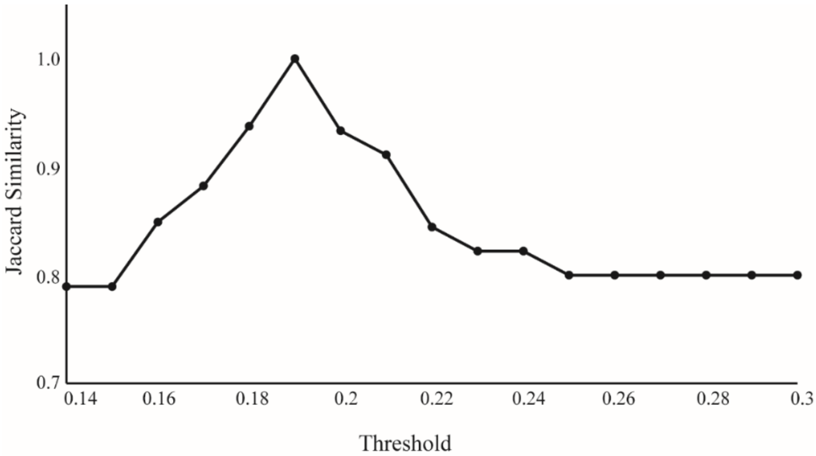

The Jaccard similarity is a statistical measure that quantifies the similarity and diversity between two sets, **A** and **B**. It is defined as the ratio of the size of their intersection to the size of their union:

$$\text{Jaccard similarity} = {|\mathbf{A} \cap \mathbf{B}|}/{|\mathbf{A} \cup \mathbf{B}|}.$$

2. Supplementary Phylogenetic Comparison of IBV Strains

The phylogenetic tree of IBVs shown below was constructed using IQ-TREE with the TIM3 substitutional model. The existing IBV classification is represented in the inner circle, while the IQ-TREE classification is depicted in the outer circle. The IBV strains in the tree are color-coded following the same scheme as Figure 2. By employing this character-based classification approach on the dataset of 311 IBVs, we categorized them into 14 genotypes and 4 outliers. Additionally, the GI genotype can be divided into 22 lineages and 2 outliers. This classification closely aligns with the existing IBV classification and the results of MSClustering at a local level. However, the overall structure of its phylogenetic tree differs significantly. In particular, the predicted tree generated by IQ-TREE includes an additional 8 branches covering genotypes GVII to GVXIV. In this character-based classification, the breakdown of GI corresponds with the established IBV classification, except for the absence of lineages 7, 19, 22, 24-26, 28, and 29, which are inaccurately positioned within the branches at the upper circle. These discrepancies arise due to the heuristic search used to construct the phylogenetic tree, which may not always be entirely accurate.

IQ-TREE is a widely-used software tool for phylogenetic analysis in molecular biology, valued for its accuracy, speed, and capacity to handle large datasets. However, our comparison suggests that MSClustering yields more reliable results. We conducted tests on an Intel® Core(TM) i9-11900F @ 2.50GHz CPU, where MSClustering processed the dataset in 1.38 seconds (including 1.34 seconds for distance matrix calculation and 0.04 seconds for clustering), while IQ-TREE took 1656.25 seconds to find the best phylogenetic tree. Additionally, MSClustering provides visualizable phylogenetic networks and clustering results at different resolution levels (MSC levels 1-4), while IQ-TREE needs extra software for visualization and clustering outcomes.

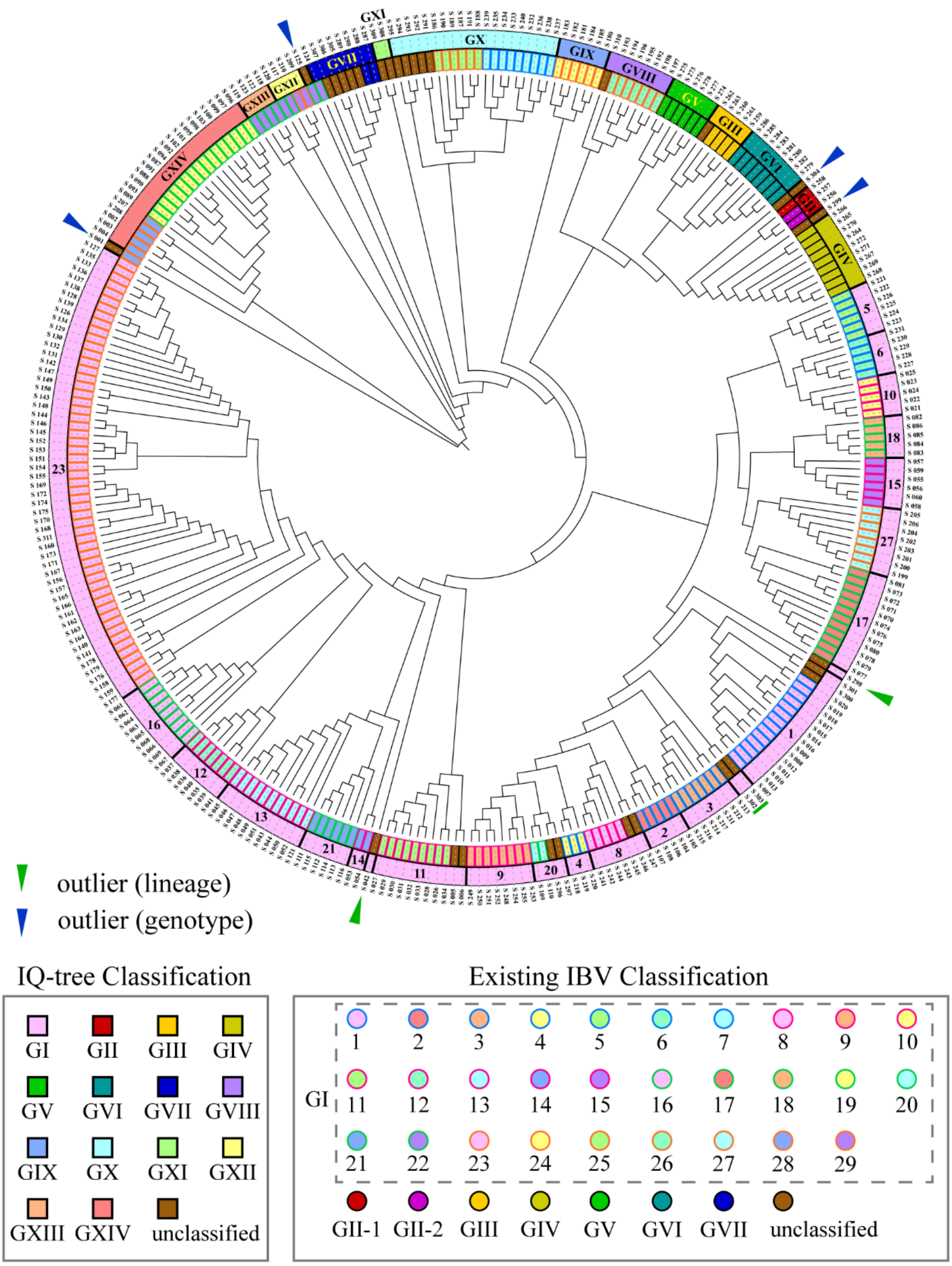

outlier (lineage)
outlier (genotype)
IQ-tree Classification
GI GII GIII GIV
GV GVI GVII GVIII
GIX GX GXI GXII
GXIII GXIV unclassified
Existing IBV Classification
GI
1 2 3 4 5 6 7 8 9 10
11 12 13 14 15 16 17 18 19 20
21 22 23 24 25 26 27 28 29
GII-1 GII-2 GIII GIV GV GVI GVII unclassified

3. Supplementary Tables

Table S1: The clustering of 311 IBV strains at levels 3 and 4 from MSClustering.

ID	NCBI_ID	MSC Levels L4	MSC Levels L3	Host	Location	Year	Length (nt)	Established Classification
S001	MF924725.1	GI	22*	Gallus gallus	South Korea	2016	1611	unclassified
S002	KU356856.1	GI	22*	chicken	China	2014	1620	unclassified
S003	KR608272.1	GI	22*	Gallus gallus	China	2016	1620	unclassified
S004	KT852992.1	GI	22*	Gallus gallus	China	2015	1620	unclassified
S005	MK581203.1	GI	outlier	Gallus gallus	Poland	1997	1623	unclassified
S006	MK581202.1	GI	outlier	Gallus gallus	Poland	1989	1620	unclassified
S007	KJ200289.1	GI	1	chicken	South Africa	2011	1600	GI-1
S008	KF809793.1	GI	1	chicken; broiler	India	2012	1611	GI-1
S009	KY626045.1	GI	1		Brazil	2016	1611	unclassified
S010	FJ888351.1	GI	1	Gallus gallus	Netherlands	1960	1611	GI-1
S011	KJ435283.1	GI	1	Gallus gallus	China (Shandong)	2011	1611	unclassified
S012	KJ425503.1	GI	1	Gallus gallus	China (Heilongjiang)	2009	1611	unclassified
S013	MK581201.1	GI	1	Gallus gallus	Poland	1989	1611	unclassified
S014	FJ904722.1	GI	1	chicken	USA (Massachusetts)	1979	1611	GI-1
S015	M95169.1	GI	1		USA (Massachusetts)	1937	1611	GI-1
S016	GQ504725.1	GI	1	chicken	USA (Massachusetts)	1941	1611	GI-1
S017	AY561711.1	GI	1		USA (Massachusetts)	1941	1609	GI-1
S018	KR902510.1	GI	1	chicken	India	2003	1611	unclassified
S019	KF411040.1	GI	1	chicken	China (Liaoning)	2011	1602	GI-1
S020	GU393333.1	GI	1	Gallus gallus	USA	1971	1590	unclassified
S021	AF151958.1	GI	10		New Zealand	2000s	1634	GI-10
S022	AF151959.1	GI	10		New Zealand	2000s	1634	GI-10
S023	AF151955.1	GI	10		New Zealand	1970s	1634	GI-10
S024	AF151956.1	GI	10		New Zealand	2000s	1634	GI-10
S025	AF151954.1	GI	10		New Zealand	1970s	1634	GI-10
S026	JX182787.1	GI	11	Gallus gallus	Brazil	1983	1609	GI-11
S027	JX182777.1	GI	11	Gallus gallus	Brazil	2008	1575	GI-11
S028	MF421320.1	GI	11	Gallus gallus	Uruguay	2011	1632	unclassified
S029	KY626044.1	GI	11		Brazil	2016	1632	unclassified
S030	MG913342.1	GI	11	Gallus gallus	Brazil	2013	1632	unclassified
S031	JX182783.1	GI	11	Gallus gallus	Brazil	2009	1612	GI-11
S032	GU393339.1	GI	11	Gallus gallus	Brazil	1984	1616	GI-11
S033	JX182775.1	GI	11	Gallus gallus	Brazil	1975	1609	GI-11
S034	JX182785.1	GI	11	Gallus gallus	Brazil	1983	1600	GI-11

S035	X52084.1	GI	12		Netherlands	1978	1614	GI-12
S036	FN182276.1	GI	12		Nigeria	2006	1613	GI-12
S037	X15832.1	GI	12		Netherlands	1979	1614	GI-12
S038	MH021175.1	GI	12		Netherlands	1979	1614	unclassified
S039	M21969.1	GI	12		Netherlands	1979	1613	GI-12
S040	X04723.1	GI	12		United Kingdom	1982	1614	GI-12
S041	AJ441314.1	GI	12		Russia (Sverdlovsk)	2002	1616	GI-12
S042	AJ440783.1	GI	outlier		Russia (Kaluga)	1999	1631	UV-
S043	AJ618985.1	GI	13		United Kingdom	1985	1616	GI-13
S044	MK581204.1	GI	13	Gallus gallus	Poland	1997	1617	unclassified
S045	JQ739375.1	GI	13	chicken	China (Shandong)	2011	1617	GI-13
S046	MGI97727.1	GI	13	chicken	China (Guangdong)	2016	1617	unclassified
S047	KP118892.1	GI	13	Gallus gallus	China (Liaoning)	2013	1617	unclassified
S048	KP118887.1	GI	13	Gallus gallus	China (Hubei)	2014	1617	unclassified
S049	Z83975.1	GI	13		United Kingdom	1991	1616	GI-13
S050	DQ386098.1	GI	13		Spain	2000	1616	GI-13
S051	AF093795.1	GI	13		Israel	1996	1616	GI-13
S052	EU914938.1	GI	13	chicken	Morocco	1983	1614	GI-13
S053	FN182277.1	GI	14		Nigeria	2006	1613	GI-14
S054	KR231009.1	GI	14	Gallus gallus	Belgium	1984	1623	unclassified
S055	FJ807933.1	GI	15	chicken	South Korea	1995	1623	GI-15
S056	JQ977697.1	GI	15	Gallus gallus	South Korea	2008	1623	GI-15
S057	FJ807932.1	GI	15	chicken	South Korea	1986	1623	GI-15
S058	AY257068.1	GI	15		Korea	2002	1620	GI-15
S059	FJ807944.1	GI	15	chicken	South Korea	2002	1623	GI-15
S060	AY257062.1	GI	15		Korea	2001	1623	GI-15
S061	EF030995.1	GI	16		China	1997	1623	GI-16
S062	AF286302.1	GI	16		China	1996-1998	1623	GI-16
S063	AF227438.1	GI	16		China	1996-1998	1623	GI-16
S064	GU938413.1	GI	16	chicken	China (Chongqing)	2009	1623	GI-16
S065	MH878976.1	GI	16	Gallus gallus	Peru	2014	1623	GI-16
S066	AY606323.1	GI	16		Taiwan	2002	1622	GI-16
S067	KJ941019.1	GI	16		Italy	1986	1623	GI-16
S068	MF421319.1	GI	16	Gallus gallus	Uruguay	2009	1623	GI-16
S069	MG021194.1	GI	16	chicken	Italy	1996	1623	unclassified
S070	AF027509.1	GI	17		USA (California)	1991	1625	GI-17
S071	AF419315.1	GI	17		USA (California)	1988	1626	GI-17

S072	FJ904714.1	GI	17	chicken	USA (California)	1995	1622	GI-17
S073	FJ904715.1	GI	17a	chicken	USA (California)	2003	1644	unclassified
S074	AF305595.1	GI	17		USA (Pennsylvania)	1998	1626	GI-17
S075	AF419314.1	GI	17		USA (Pennsylvania)	1999	1623	GI-17
S076	AF510656.1	GI	17		USA (Alabama)	1998	1632	GI-17
S077	MN512436.1	GI	17	chicken	Canada (Eastern)	2017	1623	unclassified
S078	MN512434.1	GI	17	chicken	Canada (Eastern)	2017	1623	unclassified
S079	MN512437.1	GI	17	chicken	Canada (Eastern)	2017	1623	unclassified
S080	MK878536.1	GI	17	chicken	USA (Georgia)	2019	1623	unclassified
S081	AF509583.1	GI	17a		USA (Alabama)	1997	1632	unclassified
S082	AY296745.1	GI	18		Japan	1994	1632	GI-18
S083	KC577391.1	GI	18	chicken	China	1999	1628	GI-18
S084	KC577388.1	GI	18	chicken	China	1996	1631	GI-18
S085	AY296744.1	GI	18		Japan	1993	1634	GI-18
S086	AY296746.1	GI	18		Japan	1995	1634	GI-18
S087	MH181793.1	GI	19	chicken	China (Northeastern)	2006	1620	unclassified
S088	KC577395.1	GI	19	chicken	China (Henan)	1993	1619	GI-19
S089	MGVII38155.1	GI	19	Gallus gallus (chicken)	Malaysia	2015	1620	unclassified
S090	AY189157.1	GI	19		China	2005	1617	GI-19
S091	AY043312.1	GI	19		China (Beijing)	1996	1620	GI-19
S092	GQ258308.1	GI	19	chicken	China	2008	1620	GI-19
S093	AF193423.1	GI	19		China (Qingdao)	1999	1620	GI-19
S094	KU317090.1	GI	19	Gallus gallus	China	2011	1620	unclassified
S095	MK581205.1	GI	19	Gallus gallus	Poland	2004	1620	unclassified
S096	KP662631.1	GI	19	Gallus gallus	South Africa	2011	1560	unclassified
S097	FN430414.1	GI	19		Africa	2009	1620	unclassified
S098	KY933089.1	GI	19	chicken	United Kingdom	2017	1620	unclassified
S099	EF079115.1	GI	19	chicken	Netherlands	2004	1620	GI-19
S100	KT886454.1	GI	19	Gallus gallus	Poland	2009	1620	unclassified
S101	DQ400359.1	GI	19		Israel	2004	1618	GI-19
S102	KU900739.1	GI	19	Gallus gallus	South Korea	2003	1620	unclassified
S103	MK581206.1	GI	19	Gallus gallus	Poland	2012	1605	unclassified
S104	GQ844988.1	GI	2	Gallus gallus	China (Guangzhou)	2006	1610	GI-2
S105	DQ070840.1	GI	2		China	2004-2006	1622	GI-2
S106	GU393336.1	GI	2	Gallus gallus	USA	1954	1629	GI-2
S107	GU361608.1	GI	2		USA (Iowa)	1956	1629	GI-2
S108	GU393337.1	GI	2	Gallus gallus	USA	1956	1629	GI-2

S109	AF349621.1	GI	20		Canada (Quebec)	1996	1628	GI-20
S110	AF349620.1	GI	20		Canada (Quebec)	1996-1999	1629	GI-20
S111	DQ901377.1	GI	21	commercial broilers	Italy	2002	1620	GI-21
S112	AJ457137.1	GI	21		Italy	1999	1619	GI-21
S113	DQ064808.1	GI	21		Spain	1998	1613	GI-21
S114	DQ064806.1	GI	21		Spain	1997	1619	GI-21
S115	DQ901376.1	GI	21		United Kingdom	2004	1620	GI-21
S116	DQ386105.1	GI	21		Spain	2004	1619	GI-21
S117	HQ848267.1	GI	22*	Gallus gallus	China (Guangxi)	2005	1632	unclassified
S118	HM245924.1	GI	22*	chicken	China (Chongqing)	2004	1626	unclassified
S119	DQ167147.1	GI	22*		China (Sichuan)	1999	1625	GI-22
S120	KC577397.1	GI	22*	chicken	China (Guangdong)	1998	1628	GI-22
S121	KP118894.1	GI	13	Gallus gallus	China (Guangdong)	2009	1626	unclassified
S122	GQ265940.1	GI	22*	chicken	China (South)	2008	1625	GI-22
S123	JF893452.2	GI	22*	chicken	China (Yunnan)	2005	1626	unclassified
S124	KC577382.1	GI	22*	chicken	China (Guangdong)	1997	1634	GI-22
S125	HQ018890.1	GI	22*	chicken	China (Jiangxi)	2009	1631	GI-22
S126	MF034376.1	GI	23	teal	Egypt (Qalubia)	2017	1595	GI-23
S127	MF034373.1	GI	23	teal	Egypt (Sharqia)	2017	1595	GI-23
S128	MF034375.1	GI	23	teal	Egypt (Gharbia)	2017	1595	GI-23
S129	KU238179.1	GI	23		Egypt	2015	1613	GI-23
S130	KU238178.1	GI	23		Egypt	2014	1613	GI-23
S131	KU238175.1	GI	23		Egypt	2012	1610	GI-23
S132	KU238174.1	GI	23		Egypt	2010	1613	GI-23
S133	MF034385.1	GI	23	cattle egret	Egypt (Menofia)	2017	1595	GI-23
S134	KU979008.1	GI	23		Egypt	2013	1592	GI-23
S135	MF034372.1	GI	23	house sparrow	Egypt (Sharqia)	2017	1595	GI-23
S136	MF034384.1	GI	23	teal	Egypt (Kafr El Sheikh)	2017	1595	GI-23
S137	MF034377.1	GI	23	quail	Egypt (Gharbia)	2017	1595	GI-23
S138	MF034378.1	GI	23	cattle egret	Egypt (Kafr El Sheikh)	2017	1595	GI-23
S139	MF034374.1	GI	23	teal	Egypt (Dakahlia)	2017	1595	GI-23
S140	AY279533.1	GI	23		Israel	2016	1611	GI-23
S141	AY091552.2	GI	23		Israel	1999	1611	GI-23
S142	KU979007.1	GI	23		Egypt	2012	1592	GI-23
S143	KC533682.1	GI	23	chicken	Egypt (Fayoum)	2012	1585	GI-23
S144	MN890129.1	GI	23	broiler chicken	Egypt (Qena province)	2017	1614	GI-23
S145	MN890127.1	GI	23	broiler chicken	Egypt (Qena province)	2017	1614	GI-23
S146	MN890128.1	GI	23	broiler chicken	Egypt (Qena province)	2017	1614	GI-23

S147	KU979006.1	GI	23		Egypt	2014	1592	GI-23
S148	KU238173.1	GI	23		Egypt	2011	1613	GI-23
S149	KY805846.1	GI	23	Gallus gallus	Egypt	2014	1614	GI-23
S150	KU238172.1	GI	23		Egypt	2012	1613	GI-23
S151	MN987230.1	GI	23	chicken (broiler)	Egypt	2017	1614	GI-23
S152	MN890131.1	GI	23	broiler chicken	Egypt (Qena province)	2019	1614	GI-23
S153	MN890132.1	GI	23	broiler chicken	Egypt (Qena province)	2018	1614	GI-23
S154	MN890133.1	GI	23	broiler chicken	Egypt (Qena province)	2017	1614	GI-23
S155	MN890134.1	GI	23	broiler chicken	Egypt (Qena province)	2017	1614	GI-23
S156	HM131453.1	GI	23	chicken	Israel	2016	1614	GI-23
S157	JX027070.1	GI	23	chicken	Israel	2012	1614	GI-23
S158	AF093796.1	GI	23		Israel	1998	1613	GI-23
S159	JX027069.1	GI	23	chicken	Israel	2012	1614	GI-23
S160	KY028745.1	GI	23	Gallus gallus (7-week-old broilers)	Poland	2017	1614	GI-23
S161	MF034379.1	GI	23	quail	Egypt (Sharqia)	2017	1595	GI-23
S162	JX173488.1	GI	23	Gallus gallus breed broiler	Egypt	2012	1597	GI-23
S163	MF034381.1	GI	23	cattle egret	Egypt (Kafr El Sheikh)	2017	1595	GI-23
S164	KU238171.1	GI	23		Egypt	2010	1613	GI-23
S165	EU780077.2	GI	23		Israel	2006	1597	GI-23
S166	KP259312.1	GI	23	chicken	Turkey	2012	1614	GI-23
S167	MK581207.1	GI	23	Gallus gallus	Poland	2016	1614	GI-23
S168	MF101744.1	GI	23		Romania	2017	1613	GI-23
S169	KY028748.1	GI	23	Gallus gallus (6-week-old broilers)	Poland	2017	1614	GI-23
S170	KY028746.1	GI	23	Gallus gallus (6-week-old broilers)	Poland	2016	1614	GI-23
S171	KY028743.1	GI	23	Gallus gallus (44 day-old broilers)	Poland	2016	1614	GI-23
S172	KY028747.1	GI	23	Gallus gallus (6-week-old broilers)	Poland	2016	1614	GI-23
S173	MK581208.1	GI	23	Gallus gallus	Poland	2018	1614	GI-23
S174	KY047602.1	GI	23	Gallus gallus (6 week-old broilers)	Poland	2017	1614	GI-23
S175	KY028744.1	GI	23	Gallus gallus (29-day-old broilers)	Poland	2017	1614	GI-23
S176	MGII33398.1	GI	23	chicken	Iran	2015	1620	GI-23
S177	KU238176.1	GI	23		Iran	2008	1616	GI-23

S178	MG013972.1	GI	23	chicken	Iran	2017	1620	GI-23
S179	MG013973.1	GI	23	chicken	Iran	2017	1623	GI-23
S180	KF809795.1	GI	24	chicken; Br parent	India	2013	1613	GI-24
S181	KF757447.1	GI	24		India	1998	1522	GI-24
S182	KF757450.1	GI	24		India	2007	1516	GI-24
S183	KF757451.1	GI	24		India	2004	1616	GI-24
S184	KF809791.1	GI	24	chicken; Br parent	India	2012	1613	GI-24
S185	KF809796.1	GI	24	chicken; broiler	India	2013	1616	GI-24
S186	EU925393.1	GI	25	chicken	USA (California)	2004	1616	GI-25
S187	EU694402.1	GI	25	broiler chicken	USA	2006	1603	GI-25
S188	KP085595.1	GI	25	Gallus gallus	USA (Georgia)	2012	1601	GI-25
S189	KM660636.1	GI	25	Gallus gallus	USA (Georgia)	2010	1616	GI-25
S190	JN160805.1	GI	25	Gallus gallus	USA (Georgia)	2007	1614	GI-25
S191	KP085597.1	GI	25	Gallus gallus	USA (Georgia)	2013	1604	GI-25
S192	FN182272.1	GI	26		Niger	2007	1610	GI-26
S193	FN430415.1	GI	26		Nigeria	2006	1614	unclassified
S194	FN182266.1	GI	26		Nigeria	2007	1610	GI-26
S195	FN182268.1	GI	26		Nigeria	2007	1610	GI-26
S196	FN182243.1	GI	26		Nigeria	2006	1610	GI-26
S197	FN182270.1	GI	26		Nigeria	2006	1610	GI-26
S198	FN182269.1	GI	26		Nigeria	2006	1610	GI-26
S199	MN599049.1	GI	27	broiler	USA (Georgia)	2015	1632	unclassified
S200	GU301925.1	GI	27	commercial broiler	USA (Georgia)	2008	1629	GI-27
S201	GU437858.1	GI	27	36 day-old broiler chicken	USA (NE Georgia)	2008	1632	GI-27
S202	GU437864.1	GI	27	sentinal SPF chicken	USA (NE Georgia)	2009	1632	GI-27
S203	KM660631.2	GI	27	Gallus gallus	USA (Georgia)	2010	1619	GI-27
S204	KM660634.1	GI	27	Gallus gallus	USA (Georgia)	2012	1632	GI-27
S205	AY027541.1	GI	outlier		USA (New York)	2016	1632	unclassified
S206	KM660635.2	GI	27	Gallus gallus	USA (Georgia)	2013	1632	GI-27
S207	KX640829.1	GI	22*	Gallus gallus	China (Guangxi)	2011	1620	GI-28
S208	JX291989.1	GI	22*	chicken	China (Guangxi)	2011	1616	GI-28
S209	KY407556.1	GI	22*	Gallus gallus	China	2014	1629	GI-29
S210	KY407558.1	GI	22*	Gallus gallus	China	2014	1629	GI-29
S211	AF352831.1	GI	3		Mexico	1996	1619	GI-3
S212	AF520606.1	GI	3		USA (Iowa)	1999	1629	GI-3
S213	AY789947.1	GI	3		USA (Pennsylvania)	1998	1629	GI-3
S214	L14069.1	GI	3		USA (Delmarva)	1960	1629	GI-3
S215	L14070.1	GI	3		USA (Delmarva)	1964	1629	GI-3

S216	GU393334.1	GI	3	Gallus gallus	USA	1960	1629	unclassified
S217	GQ229245.1	GI	3	chicken	Taiwan	2006	1628	GI-3
S218	KC577394.1	GI	4	chicken	China (Guangxi)	1998	1628	GI-4
S219	AY251816.1	GI	4		China (Guangxi)	2016	1629	GI-4
S220	L18988.1	GI	4		USA (Wisconsin)	1962	1629	GI-4
S221	U29453.1	GI	5		Australia	1962	1634	GI-5
S222	U29522.1	GI	5		Australia	1962	1631	GI-5
S223	AY775551.1	GI	5		China	1999	1632	GI-5
S224	AY839140.1	GI	5		China	2016	1631	GI-5
S225	DQ490215.1	GI	5		Australia	2002	1632	GI-5
S226	DQ490205.1	GI	5		Australia	2008	1632	GI-5
S227	AF151953.1	GI	6		New Zealand	1970s	1625	GI-6
S228	U29519.1	GI	6		Australia	1996	1625	GI-6
S229	JQ250818.1	GI	6	chicken	China	2010	1625	GI-6
S230	U29520.1	GI	6		Australia	1990	1625	GI-6
S231	U29523.1	GI	6		Australia	1975	1622	GI-6
S232	JQ764816.1	GI	7	Gallus gallus	China (Guangxi)	1988	1613	GI-7
S233	DQ646405.2	GI	7		Taiwan	2008	1620	GI-7
S234	MN128087.1	GI	7	chicken	Taiwan	1998	1620	unclassified
S235	KC478591.1	GI	7	chicken	China (Sichuan)	2012	1619	GI-7
S236	AY606322.1	GI	7		Taiwan	2002	1613	GI-7
S237	MN128088.1	GI	7	chicken	Taiwan	1995	1614	unclassified
S238	JQ739299.1	GI	7	chicken	China (Heilongjiang)	2011	1614	GI-7
S239	GQ229232.1	GI	7	chicken	Taiwan	2006	1620	GI-7
S240	AY606320.1	GI	outlier		Taiwan	1964	1616	GI-7
S241	JQ964066.1	GI	8	chicken	USA	1965	1631	GI-8
S242	JQ964070.1	GI	8	chicken	USA	1966	1631	GI-8
S243	JQ964071.1	GI	8	chicken	USA	1966	1631	GI-8
S244	JQ964067.1	GI	8	chicken	USA	1966	1631	GI-8
S245	JQ964061.1	GI	8	chicken	USA	1965	1631	GI-8
S246	AF520605.1	GI	8		USA (Wisconsin)	1998	1623	unclassified
S247	AF512342.1	GI	outlier		USA (Alabama)	2000	1623	unclassified
S248	KC577392.1	GI	9	chicken	China (Hainan)	1995	1628	GI-9
S249	AF006624.1	GI	9		USA (Arkansas)	1981	1629	GI-9
S250	JQ739363.1	GI	9	chicken	China (Shandong)	2011	1629	GI-9
S251	EU418975.1	GI	9		USA	2009	1632	unclassified
S252	GQ504720.1	GI	9	chicken	USA (Arkansas)	1981	1632	unclassified
S253	AF519573.1	GI	9		USA (Iowa)	1998	1632	GI-9
S254	AY514485.1	GI	9		USA (California)	2007	1632	GI-9

S255	DQ912831.1	GI	9		USA (California)	1999	1631	GI-9
S256	M21968.1	GII	GII		Netherlands	1984	1604	GII-1
S257	M21971.1	GII	GII		Netherlands	1979	1604	GII-1
S258	MK840961.1	GII	GII	Gallus gallus	Netherlands	2019	1602	GII-2
S259	DQ490219.1	GIII	GIII		Australia	1992	1632	GIII-1
S260	U29450.1	GIII	GIII		Australia	1988	1634	GIII-1
S261	U29521.1	GIII	GIII		Australia	1991	1631	GIII-1
S262	U29451.1	GIII	outlier		Australia	1988	1643	unclassified
S263	JN176213.1	GIII	GIII	Gallus gallus (chicken)	Australia	2008	1632	GIII-1
S264	AF317212.1	GIV	GIV		USA (New York)	2016	1612	GIV-1
S265	GU393332.1	GIV	GIV	Gallus gallus	USA	1992	1632	unclassified
S266	U77298.1	GIV	GIV		USA (Delaware)	1992	1605	GIV-1
S267	AF338719.1	GIV	GIV		USA (Georgia)	2000	1604	GIV-1
S268	EU283066.1	GIV	GIV		USA (Georgia)	2016	1611	GIV-1
S269	GQ504723.1	GIV	GIV	chicken	USA (Georgia)	1998	1601	unclassified
S270	AF274439.1	GIV	GIV		USA (Georgia)	1999	1604	GIV-1
S271	GQ504722.1	GIV	GIV	chicken	USA (Georgia)	1998	1601	unclassified
S272	AF274436.1	GIV	GIV		USA (Arkansas)	1997	1601	GIV-1
S273	FJ235191.1	GV	GV	Gallus gallus	Australia	2007	1494	GV-1
S274	FJ235194.1	GV	GV	Gallus gallus	Australia	2003	1494	GV-1
S275	JX018208.1	GV	GV	Gallus gallus	Australia	2008	1569	GV-1
S276	DQ059618.1	GV	GV		Australia	2003	1613	GV-1
S277	DQ059620.1	GV	GV		Australia	2003	1613	GV-1
S278	DQ059619.1	GV	GV		Australia	2003	1613	GV-1
S279	GQ265948.1	GVI	GVI	chicken	China (South)	2007	1637	GVI-1
S280	MN096598.1	GVI	GVI	chicken	China (Yunnan)	2016	1635	unclassified
S281	JX292013.1	GVI	GVI	chicken	China (Guangxi)	2009	1637	GVI-1
S282	KU361188.1	GVI	GVI	chicken	China	2014	1638	unclassified
S283	KF007209.1	GVI	GVI	chicken	China	2012	1637	GVI-1
S284	JF804687.1	GVI	GVI	chicken	South Korea	2009	1610	GVI-1
S285	JF804677.1	GVI	GVI	chicken	South Korea	2010	1610	GVI-1
S286	JF804680.1	GVI	GVI	chicken	South Korea	2009	1610	GVI-1
S287	KM365468.1	GVII	GVII	chicken	China (Guangxi)	2013	1614	GVII-1
S288	MH924835.1	GVII	GVII	Gallus	China	2017	1614	GVII-1
S289	GQ258302.1	GI	outlier	chicken	China (Anhui)	2008	1614	unclassified
S290	AY257066.1	outlier			Korea	2002	1607	unclassified
S291	EU589323.1	GI	36		Thailand	2016	1613	unclassified
S292	EU637854.1	GI	36		China (Shandong)	2005	1614	unclassified

S293	EU086600.1	GI	30	chicken	Malaysia	1995	1617	unclassified
S294	MGVII38154.1	GI	30	Gallus gallus (chicken)	Malaysia	2014	1614	unclassified
S295	GQ906705.1	GI	30	chicken	Thailand	1998	1617	unclassified
S296	AF391158.1	GI	34		USA	2016	1628	unclassified
S297	AY007235.1	GI	34		USA	2016	1628	unclassified
S298	X64737.1	GI	outlier		United Kingdom	1967	1631	unclassified
S299	AF288467.1	outlier			Mexico	2016	1616	unclassified
S300	AF218852.1	GI	33		USA	1940	1628	unclassified
S301	AF391157.1	GI	33		USA	1940	1628	unclassified
S302	AF391154.1	GI	35		USA	1940	1610	unclassified
S303	AF218851.1	GI	35		USA	1940	1610	unclassified
S304	AY789942.1	outlier			USA (Pennsylvania)	1998	1581	unclassified
S305	KM213963.1	GI	31	Gallus gallus	China (Jiangsu)	2013	1632	unclassified
S306	AY427819.1	GI	31		China	2003	1632	unclassified
S307	AY319651.1	GI	outlier		China	2003	1632	unclassified
S308	KC008600.1	GI	32	chicken	China (Guangxi)	1985	1626	unclassified
S309	JQ764815.1	GI	32	Gallus gallus	China (Guangxi)	1985	1625	unclassified
S310	AJ458942.1	GI	outlier		Russia (Irkutsk)	1999	1610	unclassified
S311	MF101746.1	GI	23		Romania	2017	1613	GI-23

Table S2: IQ-TREE selection criteria (BIC, AICc, and AIC) for evolutionary substitution models in IBV strains. The Top 20 models for the evolution of S1 gene are listed. The lowest scores are highlighted in red.

Evolutionary Substitution Models for IBVs

Model (S1 gene)	AIC	AICc	BIC
TIM3+F+R10	184485	185188	188026.4
TIM3+F+R10	184501.5	185204.6	188043
GTR+F+R5	184578.2	185259.2	188075.6
GTR+F+R5	184599.8	185280.9	188097.2
GTR+F+R5	184631.6	185312.7	188129
GTR+F+R5	184637.9	185318.9	188135.3
GTR+F+R5	184668.6	185349.7	188166
GTR+F+R5	184697.3	185378.4	188194.7
GTR+F+R5	184709.3	185390.4	188206.7
GTR+F+R5	184710.1	185391.2	188207.5
TIM3+F+R10	184686.6	185389.7	188228.1
TIM3+F+R10	184693.4	185396.5	188234.9
GTR+F+R5	184751	185432.1	188248.4
GTR+F+R5	184763.2	185444.3	188260.6
TIM3+F+R10	184722.9	185425.9	188264.3
GTR+F+R5	184812.5	185493.6	188309.9
GTR+F+R5	184822.4	185503.4	188319.8
GTR+F+R5	184841.3	185522.3	188338.7
GTR+F+R5	184927.8	185608.9	188425.2
GTR+F+R5	185179	185860	188676.3

Table S3: Selection criteria (log-likelihood) for 7 evolutionary substitution models in IBV strains using PhyML, conducted without bootstrap analysis. The highest probability is visually highlighted in red for clarity.

**Model**	**Log-likelihood**	**Computing Time (s)**
GTR	-91845	1688
TN93	-92113	1052
HKY85	-92153	1228
F84	-92177	995
K80	-92607	1835
JC69	-94671	1127
F81	-94811	1148

Table S4: Predicted recombination events among coronaviruses using RDP5, with a *P*-value threshold set at 5×10^{-4} derived from RDP. Events involving unknown parents are excluded from the listing.

Position		Sequence ID			Statistical tests (p-value)				
Begin	End	Recombinant	Minor parent	Major parent	RDP	GENECONV	Bootscan	Maxchi	Chimaera
1*	766	S121	S122	S049	7.66E-31	5.78E-27	2.82E-23	2.10E-23	4.81E-24
			S118	S045					
			S123	S047					
				S048					
				S051					
18*	792	^S306	S307	S289	7.71E-30	6.29E-30	5.47E-24	3.44E-24	4.98E-25
		S305							
1119	1810*	^S158	Unknown	S166	1.78E-24	3.13E-22	1.79E-23	6.10E-16	4.84E-17
		S159	(S140)	S156					
			Unknown	S157					
			(S141)	S160					
				S161					
				S162					
				S163					
				S164					
				S165					
				S167					
				S168					
				S169					
				S170					
				S171					
				S172					
				S173					
				S174					
				S175					
				S176					
				S178					
				S179					
				S311					
1*	722	S003	S093	S119	1.11E-23	1.90E-20	9.59E-18	1.16E-17	3.27E-17
		S002	S088	S118					
		S004	S090	S122					

		S207		S123					
1602	1811*	^S046	Unknown (S183)	S049<br>S048	2.81E-22	7.40E-22	2.33E-20	2.52E-05	2.44E-05
1571	1810*	S066	S233<br>S232<br>S234<br>S235<br>S236<br>S237<br>S238<br>S239	S061<br>S062<br>S063<br>S064	4.68E-21	4.77E-16	1.05E-19	7.94E-08	4.62E-07
120	863	^S307	S124	S092<br>S087<br>S089<br>S090<br>S091<br>S093<br>S094<br>S095<br>S096<br>S097<br>S098<br>S099<br>S100<br>S101<br>S102<br>S103	6.52E-21	1.96E-17	7.06E-17	1.11E-13	4.00E-14
802	1016	^S050	S112	S044	1.23E-19	4.34E-18	6.40E-12	1.36E-06	1.21E-06
856	1792*	^S143	S142<br>S131	S159<br>S158	1.15E-19	1.42E-16	6.24E-16	3.05E-20	1.99E-15
1119	1810*	^S177	Unknown (S141)<br>Unknown (S140)	S176<br>S156<br>S157<br>S160<br>S161<br>S162<br>S164	9.11E-19	1.19E-18	2.08E-19	4.95E-17	3.99E-17

				S165					
				S166					
				S167					
				S168					
				S169					
				S170					
				S171					
				S172					
				S173					
				S174					
				S175					
				S178					
				S179					
				S311					
110	799	^S181	S185	S008	6.30E-18	1.89E-16	4.54E-13	8.09E-15	2.08E-15
		S182[P]		S007					
				S009					
				S010					
				S011					
				S012					
				S013					
				S014					
				S015					
				S016					
				S017					
				S018					
				S019					
				S020					
20*	726	^S095	S001	Unknown	3.60E-18	7.38E-14	3.05E-14	6.81E-12	3.14E-12
		S087	S208	(S233)					
		S088		Unknown					
		S089		(S232)					
		S090		Unknown					
		S091		(S236)					
		S092		Unknown					
		S093		(S238)					
		S094		Unknown					

		S097		(S234)					
		S098		Unknown					
		S099		(S235)					
		S100		Unknown					
		S101		(S237)					
		S102		Unknown					
		S103		(S239)					
1011	1390	^S083	S019	S082	1.40E-17	2.60E-15	1.90E-14	3.64E-08	1.42E-08
		S084	S018						
			S020						
411	628	^S176	Unknown	S156	1.02E-18	1.51E-17	2.89E-16	1.90E-10	4.38E-08
		S177	(S037)	S143					
		S178	Unknown	S147					
		S179	(S036)	S148					
			Unknown	S149					
			(S039)	S150					
			Unknown	S151					
			(S041)	S152					
			Unknown	S153					
			(S035)	S154					
			Unknown	S155					
			(S038)	S157					
			Unknown	S160					
			(S040)	S161					
				S162					
				S163					
				S164					
				S165					
				S166					
				S167					
				S168					
				S169					
				S170					
				S171					
				S172					
				S173					
				S174					

				S175					
				S311					
173	680	S140	Unknown	S133	2.49E-16	8.53E-13	6.86E-11	4.69E-16	7.28E-14
		S141	(S037)	S126					
			Unknown	S127					
			(S036)	S128					
			Unknown	S129					
			(S039)	S130					
			Unknown	S131					
			(S035)	S132					
			Unknown	S134					
			(S038)	S135					
			Unknown	S136					
			(S040)	S137					
				S138					
				S139					
				S142					
1577	1809*	S183	S049	S184	1.95E-15	9.62E-15	1.46E-15	8.14E-06	3.43E-06
			S047	S180					
				S185					
692	1772*	S149	S131	S144	9.19E-16	1.67E-12	2.98E-16	9.14E-15	4.92E-07
		S147		S145					
		S150		S146					
1638	1804*	^S078	S190	S080	1.52E-13	7.46E-10	1.30E-12	1.24E-04	2.02E-03
		S077							
		S079							
1054	1642	S082	S239	S086	1.92E-12	1.43E-09	4.44E-06	1.14E-11	1.27E-10
			S236	S085					
1204	1515	S210	Unknown	S117	1.16E-11	1.01E-06	8.43E-09	5.29E-11	1.79E-07
		S209	(S184)						
			Unknown (S185)						
			Unknown (S180)						
210*	443	^S131	S035	S143	8.28E-12	1.07E-06	1.44E-09	3.13E-09	3.63E-06
		S126	S036						
		S127	S037						
		S128	S038						
		S129	S039						

		S130	S040						
		S132	S041						
		S133							
		S134							
		S135							
		S136							
		S137							
		S138							
		S139							
		S142							
1176	1805*	^S292	S187	S291	4.27E-11	8.41E-08	2.58E-08	5.66E-10	3.15E-09
			S186						
			S190						
1*	732	^S119	S120	S001	1.62E-08	1.41E-07	2.63E-07	5.84E-11	3.00E-11
		S118							
		S122							
		S123							
24*	720	^S232	S240	S233	3.90E-07	3.34E-05	4.26E-04	1.05E-08	1.15E-10
				S234					
				S235					

Table Key:

~ = It is possible that this apparent recombination signal could have been caused by an evolutionary process other than recombination.

*= The actual breakpoint position is undetermined (it was most likely overprinted by a subsequent recombination event).

^ = The recombinant sequence may have been misidentified (one of the identified parents might be the recombinant)

Minor Parent = Parent contributing the smaller fraction of sequence.

Major Parent = Parent contributing the larger fraction of sequence.

Unknown = Only one parent and a recombinant need be in the alignment for a recombination event to be detectable. The sequence listed as unknown was used to infer the existance of a missing parental sequence.

4. Supplementary Figures

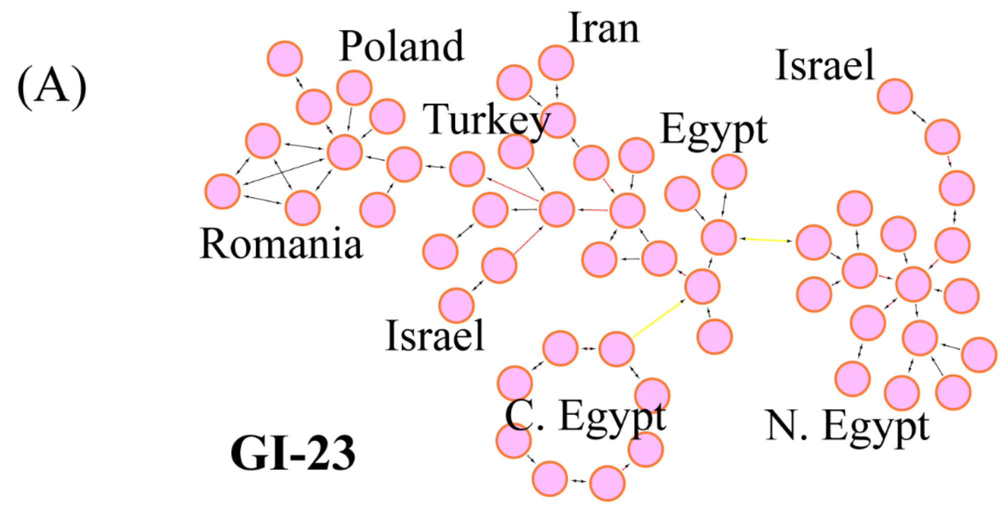

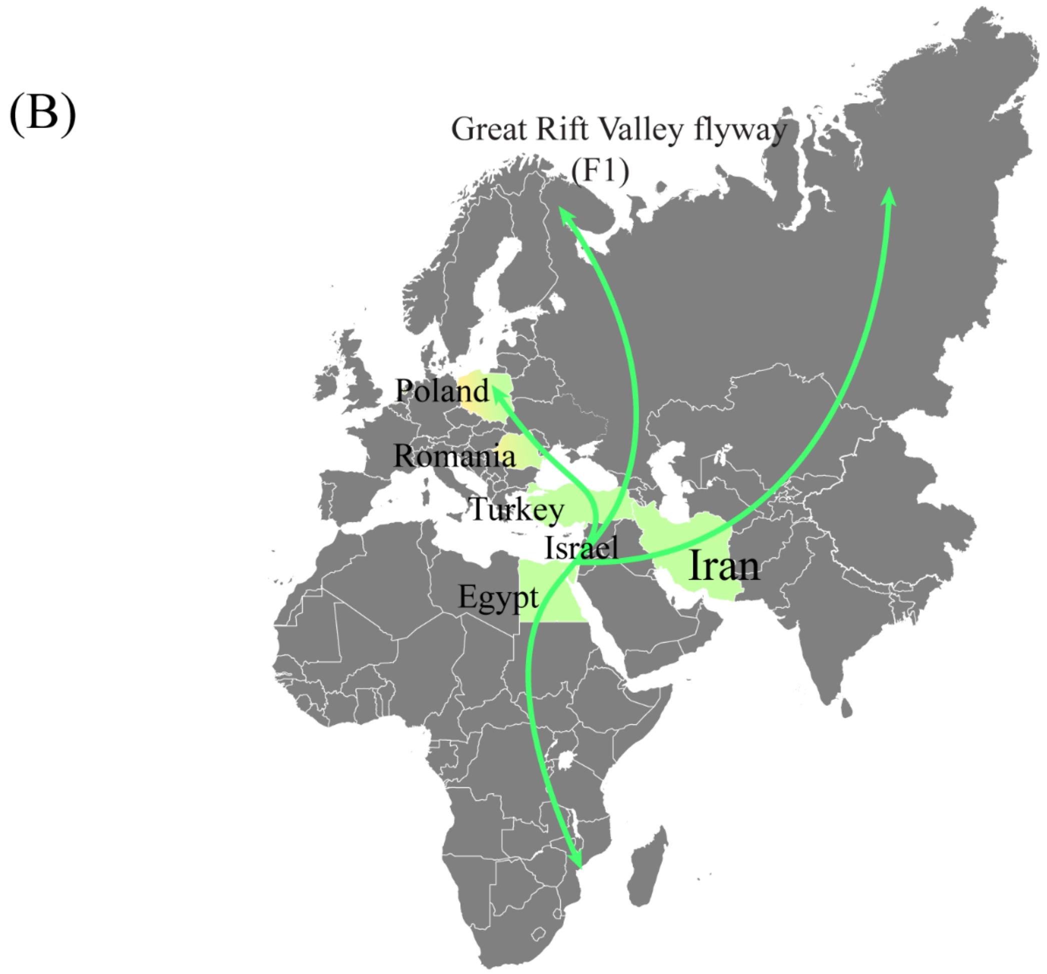

Figure S1. The phylogenetic network of GI-23 (A) and the Great Rift Valley migratory flyway F1 (B).

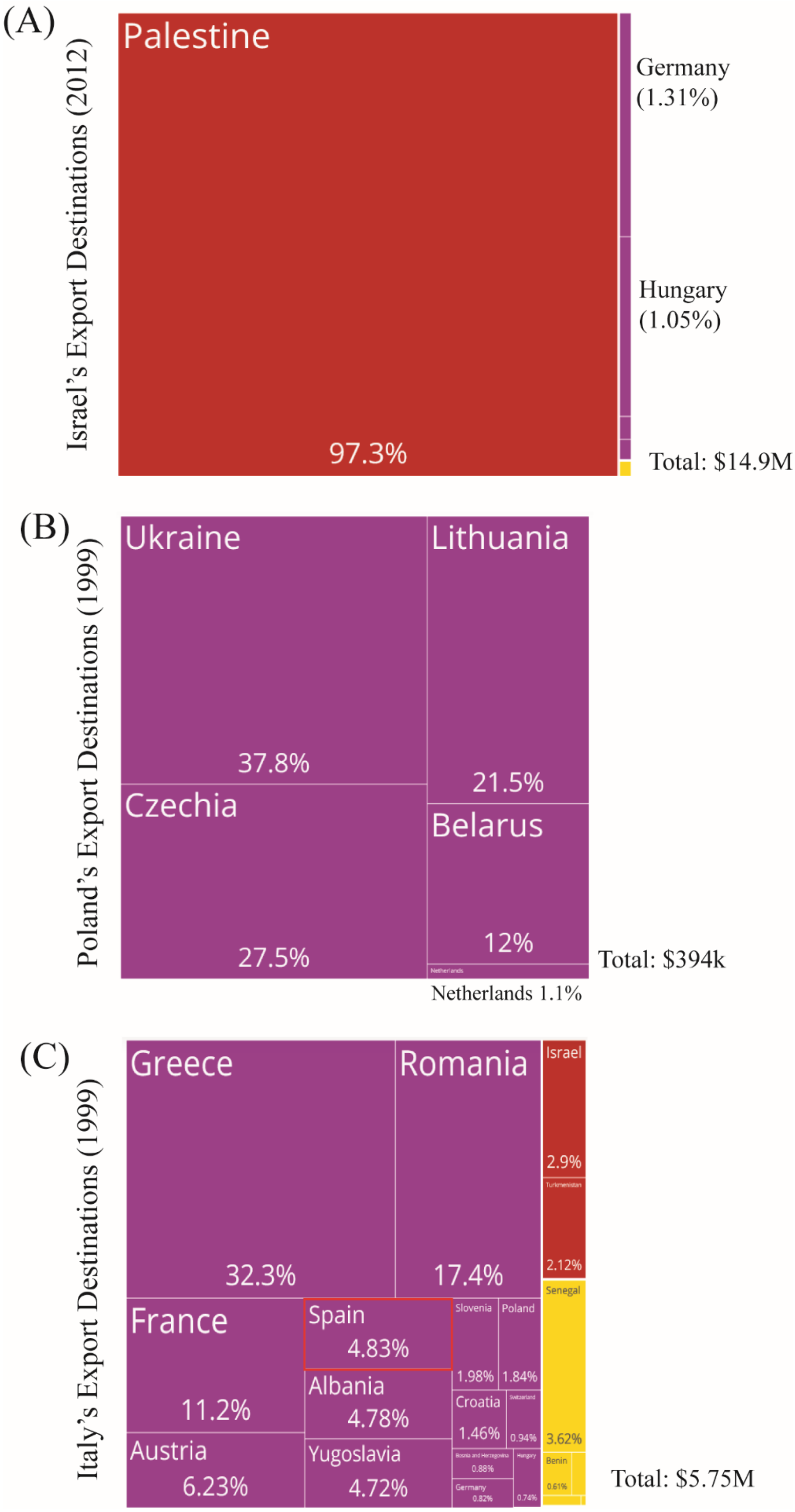

Figure S2. Poultry exports of Israel in 2012 (A), Poland in 1999 (B), and Italy in 1999 (C).

OECD. (n.d.). Poultry exports and imports data. Observatory of Economic Complexity. Retrieved July 7, 2024, from https://oec.world/en/profile/hs/poultry.
